# LISA improves statistical analysis for fMRI

**DOI:** 10.1038/s41467-018-06304-z

**Published:** 2018-10-01

**Authors:** Gabriele Lohmann, Johannes Stelzer, Eric Lacosse, Vinod J. Kumar, Karsten Mueller, Esther Kuehn, Wolfgang Grodd, Klaus Scheffler

**Affiliations:** 1 Department of Biomedical Magnetic Resonance Imaging, University Hospital Tübingen, Hoppe-Seyler-Strasse 3, 72076 Tübingen, Germany; 2 Magnetic Resonance Centre, Max-Planck-Institute for Biological Cybernetics, Max-Planck-Ring 11, 72076 Tübingen, Germany; 3 Max-Planck-Institute for Intelligent Systems, Max-Planck-Ring 4, 72076 Tübingen, Germany; 4 Methods & Development Group Nuclear Magnetic Resonance, Max-Planck-Institute for Human Cognitive and Brain Sciences, Stephanstrasse 1A, 04103 Leipzig, Germany; 5 German Center for Neurodegenerative Diseases (DZNE), Leipziger Strasse 44, 39120 Magdeburg, Germany; 6 Center for Behavioral Brain Sciences (CBBS), 30120 Magdeburg, Germany; 7 Department of Neurology, Max-Planck-Institute for Human Cognitive and Brain Sciences, Stephanstrasse 1A, 04103 Leipzig, Germany

## Abstract

One of the principal goals in functional magnetic resonance imaging (fMRI) is the detection of local activation in the human brain. However, lack of statistical power and inflated false positive rates have recently been identified as major problems in this regard. Here, we propose a non-parametric and threshold-free framework called LISA to address this demand. It uses a non-linear filter for incorporating spatial context without sacrificing spatial precision. Multiple comparison correction is achieved by controlling the false discovery rate in the filtered maps. Compared to widely used other methods, it shows a boost in statistical power and allows to find small activation areas that have previously evaded detection. The spatial sensitivity of LISA makes it especially suitable for the analysis of high-resolution fMRI data acquired at ultrahigh field (≥7 Tesla).

## Abstract (web summary)

Functional magnetic resonance imaging (fMRI) is a powerful technique for measuring human brain activity, but the statistical analysis of fMRI data can be difficult. Here, the authors introduce a new fMRI analysis tool, LISA, which provides increased statistical power compared to existing techniques.

## Introduction

A principal goal in functional magnetic resonance imaging (fMRI) is the localization of brain activity associated with regional changes in deoxyhemoglobin concentration^1^. Statistical inference is a crucial part of fMRI data analysis that is needed in order to separate signal from noise. Due to the high dimensionality of the data, this is a challenging task for which a wide range of methods have been employed over the past 25 years, e.g., refs. ^2–7^.

The aim of statistical methods is to distinguish true brain activations from false alarms so that both false negative and false positive rates are kept at bay. Recent publications argue that brain mapping studies show deficits in both respects. In fact, several of the most widely used software packages were found to produce inflated false positive rates meaning that some of the insights about human brain function gained with the help of neuroimaging may in fact be artifactual^8^. For more about this controversy, see refs. ^9–11^.

On the other hand, failure to detect true activations due to lack of statistical power may be an even more pressing problem^12–15^. Increasing sample sizes improves power, but the acquisition of large samples is expensive and not always practicable. Median sample sizes in group studies have steadily increased since 1995, but in 2015 they are still below 30^7^. Thus, new analysis methods with improved sensitivity are urgently needed so that better results are obtainable using reasonable sample sizes.

In this paper, we introduce a new method of statistical inference in fMRI. We call it LISA because it is inspired by the concept of Local Indicators of Spatial Association used in geographical information systems^16^. Its main innovation is the way in which spatial information is used for improving statistical power and preserving spatial precision. LISA can be applied to many different types of input maps, including standard group data, single subject activation maps measured at high spatial resolutions, results obtained by multivariate pattern analysis (MVPA), and many more.

The procedure for deriving brain activation maps generally starts out with a general linear model (GLM) regression in which voxel time courses are correlated with a predefined hemodynamic response model^2,17,18^. This results in a contrast map in which every voxel contains the difference between two or more regression intercepts representing effect strengths of various experimental conditions. For a review of standard fMRI data analysis, see e.g., ref. ^19^.

Obtaining a map of brain activations from these initial contrast maps is a difficult task because it requires statistical tests to be performed in thousands of voxels simultaneously so that many false positives are to be expected. This is known as the multiple comparison problem^20,21^. For example, if the number of voxels covering the brain is 100,000 and a significance threshold of *p* < 0.05 is used, we may expect around 5000 false positives. A more stringent threshold would reduce the number of false positives but would also lead to a loss in statistical power.

Note that univariate null hypothesis significance tests such as the *t*-test ignore spatial information so that adjacent voxels are treated as if they were independent. However, fMRI images exhibit spatial coherence so that brain activations do not consist of isolated voxels. This fact can be exploited to correct for multiple comparisons. The various methods of statistical inference in fMRI differ primarily in the way in which this is done.

A common strategy is to reduce the number of tests by computing the test statistic not on individual voxels, but on entire activation regions where each region (also termed “cluster”) is defined as a group of connected voxels with high activation levels^18,22–26^. This generally reduces the number of tests from thousands of voxels to a few dozen clusters. Larger clusters are less likely to arise by chance so cluster size can be efficiently used as a test statistic. About 75% of all published fMRI studies follow this approach^27^.

One of the main problems in approaches based on cluster size is that an initial cluster-defining threshold (CDT) is required, the exact choice of which can be highly problematic^27^. Very stringent thresholds may eliminate relevant brain activations right away and hence lead to a loss in statistical power. Very liberal thresholds on the other hand may produce inflated false positive rates^8^ and cause adjacent regions to merge into large agglomerates that lack spatial specificity. A threshold-free method called “threshold-free cluster enhancement” (TFCE) has therefore been proposed to avoid the problems of thresholding^28^. However, TFCE may suffer from limitations of low spatial specificity when significant clusters are large^27^.

A widely used cluster-size inference method is based on the Gauss Random Field (GRF) model^18,23^. Implementations of the GRF approach are available in several major software packages^29–31^. The main problem with GRF is that it requires spatial smoothing during preprocessing in order to meet the underlying assumption of Gaussianity^32^. According to standard guidelines, the kernel size needed for Gaussian spatial smoothing is 2–3 times the voxel size ^19,33^, so that the GRF can only be applied at the cost of a massive loss of spatial precision. With the advent of new scanning technology at ≥7 T, neuroscience is now equipped with much better imaging data acquired at very high spatial resolutions that should clearly not be sacrificed just to fulfill the needs of statistical inference^15,34^. A non-parametric and data-driven way to estimate a null model is to use random permutations of subjects or task labels^5,35–37^. These methods are not limited by Gaussianity requirements.

Statistical inference methods also differ in the type of error they control. The two most common ones are the family-wise error rate (FWER)^38^ and the false discovery rate (FDR)^20,39–43^. The FDR is the percentage of false positives among all reported activations whereas FWER is the probability of obtaining one or more false positives.

In the following, we present a new algorithm called LISA for multiple comparison correction in MRI activation maps. The main idea is to incorporate spatial context via a non-linear filter so that spatial precision is preserved and thresholding for cluster-formation is avoided. Multiple comparison correction is achieved by a voxelwise control of the FDR in the filtered maps.

## Results

### Overview of the method

LISA can be applied in several different scenarios. The first is a group-level analysis involving a onesample test across a group of contrast maps. The second scenario is a single subject analysis involving a test based on only one data set Finally, LISA can be used in a generic form that is independent of the GLM. In the following, we describe each of these points in detail. For a pseudocode, see Supplementary Note 1.

### Group-level analysis

In this setting, the input into LISA is a group of real-valued 3D images defined on a voxel grid. Typically, they represent contrast maps obtained via a first-level GLM analysis. The output of LISA is a 3D image in which every voxel has a value in [0, 1] representing its FDR. FDR controls the proportion of falsely rejected null hypotheses. Here, the null hypothesis for every voxel is that the group mean is not positive. In the following, we give a detailed description of each step of this procedure.

### Obtaining an initial *z*-map

Group-level inference in LISA begins with a voxelwise onesample *t*-test across a group of contrast maps that typically originate from a GLM-based regression. This initial step is a standard procedure shared by many other methods. It yields a map of *z*-scores (the “*z*-map”) uncorrected for multiple comparisons in which each voxel has a standardized *z*-score.

### Incorporating spatial context

**Fig. 1:**
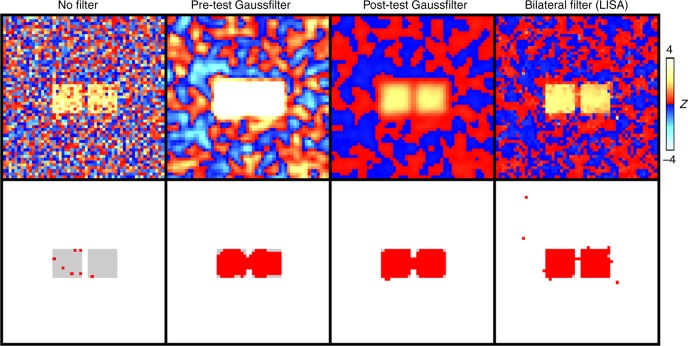
Motivating LISA. The top row shows *z*-maps obtained by applying a voxelwise onesample *t*-test applied to 20 randomly generated images. The maps were computed with either no spatial filtering, or spatial filtering before versus after applying the *t*-test. The bottom row shows the same results thresholded so that the false discovery rate was 0.05 with the simulated signal as an underlay in light grey. Here we scaled the filtered and unfiltered *z*-values to the same range. Note however, that after filtering the *z*-values are no longer standardized so that their interpretation differs. The true positives rates were 0.035 (no filter), 0.89 (pre-test Gaussfilter), 0.97 (post-test Gaussfilter), 0.99 (post-test bilateral filter). Applying the Gaussfilter prior to the *t*-test diminishes spatial accuracy and yields a poor true positive rate. A better result is achieved by applying the Gaussian filter after the *t*-test. The post-test bilateral filter produces the best results in terms of both spatial accuracy and statistical power. Therefore, we propose this approach for our new method “LISA”

The next step in the LISA analysis chain is to apply an edge-preserving spatial filter to the *z*-map. The effect of this filter is to enhance the signal and suppress noise while preserving spatial accuracy. This step is quite unconventional because the standard procedure is to apply spatial filtering during preprocessing, but not as a postprocessing of the *z*-map. The unusual order in which spatial filtering is applied here offers distinct advantages, see Fig. 1 for an illustration.

A large range of adaptive filters exist that might be used in this context^44–48^. In the experiments reported below, we have used the bilateral filter because it is widely used and well known^49–51^, see also Supplementary Figure 25. The SUSAN filter^52^ is very similar and can therefore be expected to yield comparable results. Note that the main innovation in LISA is the order in which adaptive spatial filtering is applied, not the specific type of filter that is used.

The bilateral filter uses a product of two Gaussian kernels where one kernel penalizes spatial distance while the other penalizes discrepancies in voxel values. More precisely, let *i* denote some voxel position, Ω_*i*_ its local neighbourhood, and *z*_*i*_ its value in the map of uncorrected values. Then the filtered value *λ*_*i*_ is defined as

1 $$\lambda _i = \frac{1}{{W_i}}\mathop {\sum}\limits_{j\, \in \,{\mathrm{\Omega }}_i} z_if_r(||z_i - z_j||){\kern 1pt} g_s(d(i,\,j))$$

with *f*_*r*_ the range kernel for voxel intensities, *g*_*s*_ a spatial kernel for weighting differences in voxel coordinates, and *d* the Euclidean distance between voxels *i* and *j*. The normalizing factor *W*_*i*_ is defined as $\mathop {\sum}\nolimits_{j\, \in \,{\mathrm{\Omega }}_i} f_r(||z_i - z_j||){\kern 1pt} g_s(d(i,\,j))$. As customary, we use Gaussian kernels for *f*_*r*_ and *g*_*s*_ with *f*_*r*_(*x*) = exp(−*x*^2^/*σ*_*r*_) and *g*_*s*_(*x*) = exp(−*x*^2^/*σ*_*s*_).

In the experiments reported below, we used the same parameter settings throughout, namely *σ*_*s*_ = 2.0 voxels, *σ*_*r*_ = 2.0 and a spherical neighbourhood Ω_*i*_ with a radius of two voxels comprising 117 voxels. The parameter *σ*_*r*_ depends on the range of values present in the input and permuted maps. We therefore scale all input maps by the standard deviation of the values of the first 30 permuted maps. Bilateral filtering can be applied iteratively. In all our experiments, we used two iterations.

Special consideration must be given to voxels for which part of their local neighbourhood is outside of the brain. We use a threshold to separate brain from non-brain tissue. If the voxel is inside the brain, but more than half of its neighbourhood is outside, we use a median filter in a smaller 18-neighbourhood provided at least half of this smaller neighbourhood is inside the brain. Otherwise the voxel is discarded.

### Controlling the false discovery rate

The final stage of the analysis chain in LISA is to control the FDR of the filtered *z*-map. Here, we use a non-standard approach for estimating FDR because the classical algorithm by Benjamini and Hochberg^39,53^ is not applicable in our case because it expects *p*-values or standardized *z*-values as input. This requirement is violated here because of the spatial filter applied to the *z*-map.

Instead, we propose to control FDR using a Bayesian two-component mixture model in which the hypothesis to be tested has a prior probability *p*_0_ of being null and *p*_1_ = 1 − *p*_0_ of being non-null with corresponding density functions *f*_0_(*λ*) and *f*_1_(*λ*)^54,39,55^, Let *F*_0_(*λ*) and *F*_1_(*λ*) be their left-sided cumulative distribution functions and *F*_*z*_(*λ*) their mixture, so that

2 $$F_z(\lambda ) = p_0F_0(\lambda ) + p_1F_1(\lambda ).$$

and hence

3 $$Fdr(\lambda ) = p_0F_0(\lambda )/F_z(\lambda )$$

Since the estimation of *p*_0_ is not trivial, we simply assume *p*_0_ = 1 which is the most conservative choice.

An estimate of *F*_*z*_ is obtained from the histogram of the filtered *z*-map. The null distribution *F*_0_ is derived from random permutations of subjects (i.e., contrast maps). In each permutation, contrast maps are randomly selected with probability 0.5 and the signs of all values in the selected maps are switched. Spatial autocorrelations remain intact in these permutations because we switch all signs in the entire contrast map simultaneously. In our experiments, we used 5000 such permutations. Next, a *t*-test is performed and the bilateral filter is applied to the resulting permuted *z*-map. From the histogram of these permuted maps, we obtain an estimate of *F*_0_, resulting in an FDR score for every voxel, see Eq. (3).

Note that permutation testing requires that under the null hypothesis data can be exchanged without significantly affecting the results. As customary in fMRI, we assume here that subjects are exchangeable. See refs. ^35,36,56^ for more information about this issue.

Note that this approach can be easily transferred to a two-sample test scenario. In this case, the input into LISA is two groups of contrast maps. In each random permutation, every contrast map is randomly assigned to one of the two groups. The rest of the analysis proceeds as before.

### Single subject analysis

In a single subject setting, the input into LISA is a 4D fMRI time series data set together with information about the experimental design, i.e., onsets, durations, and task labels of all trials. Furthermore, a contrast vector must be specified indicating which experimental conditions are to be contrasted. The input data set must have been preprocessed using some standard preprocessing pipeline. The experimental design must be randomized and must ensure exchangeability across trials. The output of LISA is a 3D image in which every voxel has a value in [0, 1] representing its FDR score where FDR controls the proportion of falsely rejected null hypotheses. Here, the null hypothesis is that the user-defined contrast is not positive. A more detailed description follows.

### Obtaining a filtered *z*-map

Single subject inference in LISA begins with a GLM-based regression that results in a single contrast map. Here, we assume that each experimental condition (“task”) is represented by multiple trials that are spread randomly over the duration of the experiment. The contrast map is converted into a map of standardized *z*-scores uncorrected for multiple comparisons (“*z*-map”) as described in ref. ^18^. As in the group-level case, a bilateral filter is subsequently applied to the *z*-map, and its histogram is used to estimate *F*_*z*_.

### Exchangeability

Special care must be taken to ensure exchangeability of the permutations. Volumes (i.e., 3D images of the brain) recorded at different timepoints, are not exchangeable because of temporal autocorrelations. Here, we propose to use random permutations of task labels that leave the temporal structure of the data intact^57^. In each random permutation, every trial receives a new task label that is randomly selected from the pool of all task labels. Note that the task labels are exchangeable provided the experimental design is properly randomized and the inter-trial distances are large enough so that the trials are independent. Subsequently, a new hemodynamic model is fitted and a *z*-map computed to which the bilateral filter is applied. In experiments with multiple runs, task labels are only permuted within the same run so that exchangeability is ensured. For more on random permutations in this setting, see refs. ^36,58^. As before, the histograms of the permuted maps are used to estimate the null distribution *F*_0_, and by Eq. (3) an FDR score is obtained for every voxel.

### Generic LISA

LISA is not limited to the scenarios listed above. A generic implementation that generalizes to other domains is also available. In its generic form, LISA expects two files as input. The first file contains a non-permuted input map. The second file contains a list of permuted maps generated with the same test statistic after applying a random permutation. LISA subjects all maps to the bilateral filter, and outputs a map with FDR scores at every voxel. For example, the generic LISA can be applied in the context of MVPA. In this case, the input into LISA may be a map of classifier weights^59^. Permuted maps are generated by shuffling the subjects or task labels and computing a weight map in every permutation. LISA will then produce a map in which each voxel has an FDR score showing the statistical significance of the feature weight.

In the following, we assess the validity and effectiveness of the LISA algorithm using large public databases and supercomputing. Specifically, we compare the reproducibility of results under repeated sampling from a large cohort of subjects and the sample sizes needed to achieve statistical significance against several widely used methods. Furthermore, we analyze the spatial precision in ultrahigh field data. To ensure the validity of LISA, we estimate the false positive rates when applied to null models, and we estimate statistical power using simulations.

### Group studies at 3 Tesla, task fMRI

We applied LISA to fMRI data acquired at 3 Tesla by the Human Connectome Project (HCP), WU-Minn Consortium^60–62^ and compared the results with those obtained by other commonly used methods. We use the HCP data as a benchmark because it is widely known and contains a large number of data sets needed for validation purposes. We focused on the motor and the emotion task, using minimally preprocessed data of 400 unrelated participants. All data sets were spatially filtered during preprocessing using a Gaussian filter with fwhm = 6 mm. In the motor task, we investigated the left-hand finger tapping condition. In the emotion task, we used the “faces minus shapes” contrast. For details, see Supplementary Methods 3.

For comparison, we selected three major software packages, namely SPM^29^, FSL^63^, and AFNI^64,65^, which cover about 80% of all published fMRI studies^31,66^. Specifically, the following methods were included in the comparison: the GRF method implemented in SPM with FWER and FDR corrections^2,17^, the TFCE method implemented in FSL^28^, and 3dttest++ implemented in AFNI^9^. For the cluster extent based methods (AFNI, SPM), we used initial cluster forming thresholds of *p* < 0.001 and *p* < 0.01. Note that the above methods—except the FDR version of SPM—correct for FWER while LISA corrects for FDR. We will discuss this issue in more detail later on.

We used group-level onesample *t*-tests ignoring the within-subject variance. For FSL-TFCE, AFNI, and LISA, we used 5000 random permutations to estimate the null distribution. Here, we report the results of SPM controlled for FWER with *p* < 0.001 as CDT, and AFNI controlled for FWER with *p* < 0.01 as CDT, because with these parameters sensitivity is maximized and the false positive rates of AFNI are not inflated. Furthermore, we report results of FSL-TFCE and LISA. The other results are listed in Supplementary Figures 3–12.

**Fig. 2:**
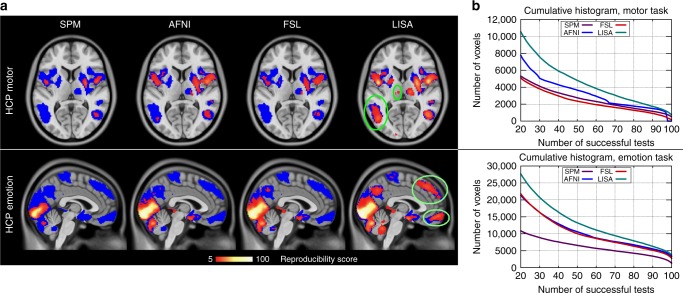
Reproducibility across 100 tests based on randomly drawn samples of size 20. The colors in the left image (panel **a**) represent reproducibility scores, i.e., the number of tests in which a given voxel consistently passed the significance threshold. The underlying blue areas show the reference map which was derived using all 400 subjects. The corresponding cumulative histograms (panel **b**) show that the reproducibility scores are considerably higher for LISA. For example, in the emotion task, LISA has detected 11,115 voxels consistently in at least 60 of the 100 tests. The corresponding numbers for the other methods are 8759 (AFNI), 8594 (FSL), and 5709 (SPM)

As a first step, we used the full cohort of all 400 subjects to obtain a reference map (Fig. 2). Specifically, we applied FSL-TFCE to all 400 subjects, and corrected for FWER < 0.01 using 5000 random permutations. High power entails an increased likelihood that statistically significant results reflect true effects so that this map can serve as a highly reliable reference^12^.

To assess reproducibility, we randomly drew 100 samples of size 20 with replacement from the cohort of 400 subjects and applied the above statistical inference procedures corrected at FWER < 0.05 or FDR < 0.05. The computation time for one sample (5000 permutations) with LISA was approximately 10 min on a standard Linux PC (16 cores). We generated maps showing the reproducibility per voxel across 100 tests (Fig. 2). When comparing the results, we found that LISA exceeded the reproducibility scores of the other methods considerably.

**Fig. 3:**
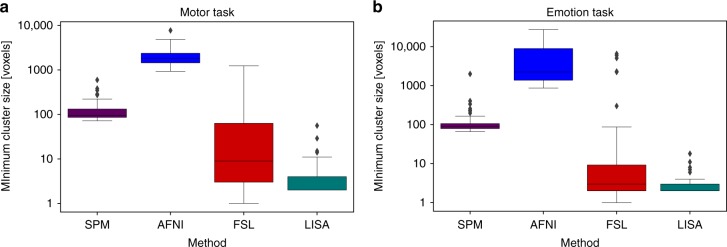
Boxplots of minimal cluster sizes averaged over all 100 tests (sample size 20). The *y*-axes are scaled logarithmically. The box spans from the first to third quartile (depicting the median as a line in the middle), the whiskers extend to 1.5 * IQR (interquartile range). The results for the motor task are shown in (panel **a**), and for the emotion task in (panel **b**). The cluster size thresholds of SPM and AFNI (3dttest++) prevent small clusters from being detected which is not a problem for FSL-TFCE and LISA. However, FSL shows a large variability in the size of the smallest cluster its finds. This is potentially harmful as cluster size plays a role in the FSL-TFCE algorithm

We also compared the clusters found by each method where clusters are defined as connected components of the resulting activation maps. We made the following observations. First, LISA not only detected more voxels, but also entire clusters that were ignored by the other methods. They are marked in Fig. 2. Second, the differences in reproducibility occur mostly in cluster centers, and not just at their peripheries (Supplementary Figures 7, 8). Third, we computed the size of the smallest clusters detected by all four methods (Fig. 3). As expected, the cluster size thresholds of SPM and AFNI prevent small clusters from being detected. For example, the median of the smallest cluster sizes found by AFNI in the emotion task was 1854 voxels = 14,832 mm^3^. For comparison, note that in healthy subjects, the entire thalamus has a volume of less than about 7700 mm^3^ ^67^. Using *p* < 0.001 as an initial CDT, much smaller clusters were detected (Supplementary Figures 3, 4, 5). However, at this more stringent threshold, AFNI produces results that are about as conservative as those of SPM. Both LISA and FSL are able to detect small clusters, but FSL shows a large variability in the size of the smallest cluster its finds, see Fig. 3.

**Fig. 4:**
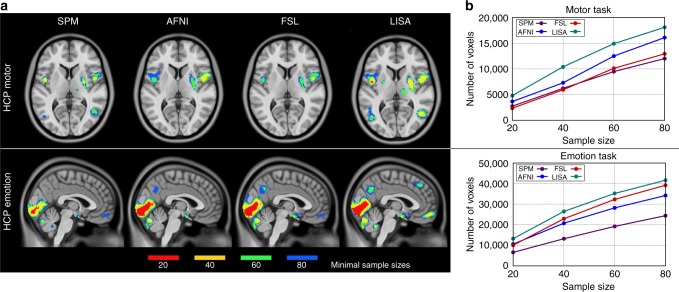
Minimal sample sizes needed for detection. The colors indicate the minimal sample sizes needed in order to detect an activation with a reasonable chance of success, i.e., in at least 50 of 100 tests (panel **a**). The corresponding histograms (panel **b**) show the number of voxels that passed the significance thresholds in at least 50 of the 100 tests for each of four different sample sizes. Note that LISA detects many more voxels at smaller sample sizes than the competing methods. For example, at sample size 40, the number of voxels detected in at least 50 of the 100 tests in the motor task were 5940 (FSL), 7292 (AFNI), 6217 (SPM), 10,401 (LISA)

Next, we computed minimal sample sizes that are needed to detect an activation with a reasonable chance of success which we defined to be 50 of the 100 tests (Fig. 4). For this purpose, we investigated various samples sizes, namely 20, 40, 60, and 80. We found that LISA detected more voxels, and even entire brain regions at noticeably smaller sample sizes. For example, in the motor task, LISA needed a sample size of 40 to detect 10,401 voxels in at least half of all tests. The best competitor (AFNI) needed a sample size of about 50 to achieve a similar result, while SPM needed sample sizes of almost 70. In the emotion task, LISA detected more voxels using samples of size 20 than SPM with samples of size 40.

Furthermore, we investigated the effect of the bilateral filter. We found that reproducibility declined markedly when we omitted the bilateral filter, see Supplementary Figures 23, 24. Lastly, we note that the type of error that is controlled in SPM (FWER versus FDR) made almost no difference, see Supplementary Figures 4, 6.

### Ultrahigh resolution imaging

Task-based fMRI data were acquired of a single subject at a 7 Tesla MR scanner with a spatial resolution of 1.5 mm^3^ ^68^. The participant gave written informed consent prior to the experiment, and was paid for her attendance. The study was approved by the local Ethics committee at the University of Leipzig. In this experiment, the subject was stimulated in the MR scanner at four fingers of her right hand using an MR-compatible tactile stimulator, for details see Supplementary Methods 4.

**Fig. 5:**
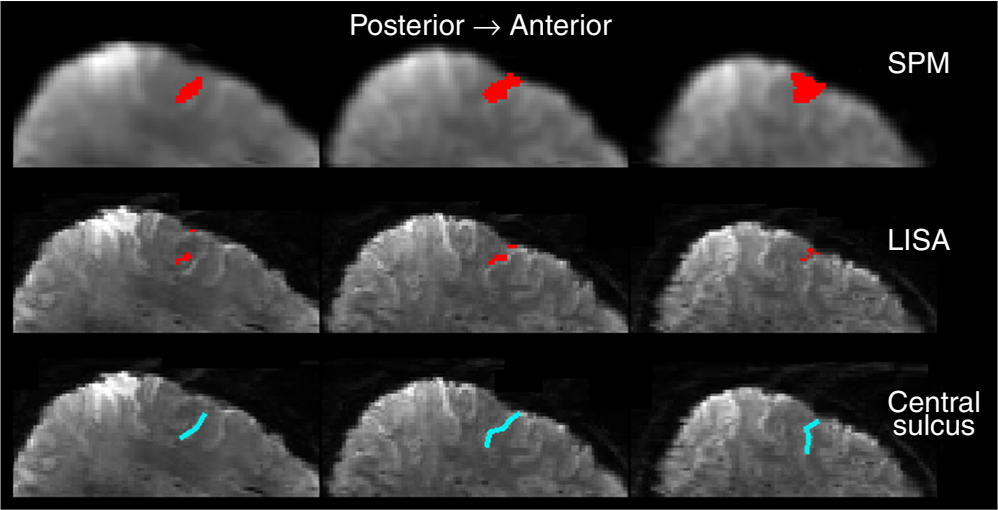
Stimulation of the index finger (single subject, 7 Tesla fMRI). The experiment is expected to activate parts of the somatosensory cortex which is located posterior to the central sulcus. The images in the bottom row show the central sulcus marked manually in four sagittal slices of the input data (not AC-PC aligned). The two top rows show the results obtained by SPM (top row, (*p* < 0.05, cluster-based FWE-corrected)) and LISA (middle row, (FDR < 0.05)) superimposed on the preprocessed input data. For SPM, a Gaussian filter was applied during preprocessing. The ensuing loss in spatial precision makes it difficult to identify the exact anatomical location of the activation. The results obtained by LISA show that the activation is indeed inside the thin cortical ribbon posterior to the central sulcus. See also Supplementary Figures 15, 16

**Fig. 6:**
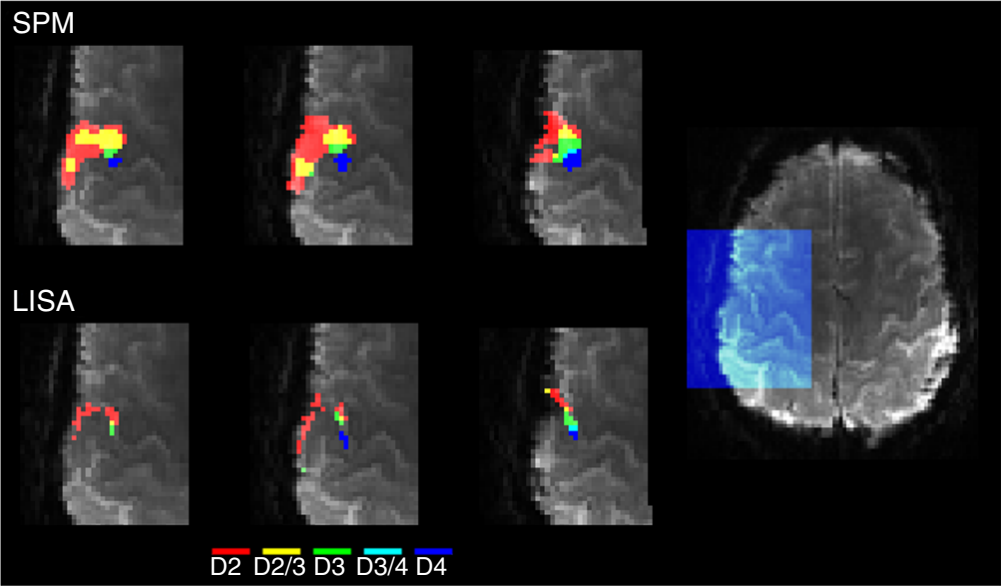
Result of a finger stimulation experiment (single subject, 7 Tesla fMRI). Four contrasts were computed. In each contrast, one finger was contrasted against all other fingers. For clarity, here we only show three of these contrasts. The colors indicate areas that show a statistically significant contrast (FDR < 0.05), index finger (D2, red), middle finger (D3, green), ring finger (D4, blue). Areas of overlap are marked in yellow (D2/3) and cyan (D3/4). Large overlaps indicate a lack of spatial specificity. Here, we only show one slice of the somatosensory area as indicated in the overview image on the right. The full images are shown in Supplementary Figures 13, 14

We compared the results obtained by LISA against those obtained by SPM. For the single subject case there are no clear guidelines for the amount of smoothing needed in SPM to ensure a valid inference. Here, we used a Gaussian kernel with fwhm = 4 mm which is consistent with the guidelines for group-level studies. We made this selection based on the report by Eklund et al. who found that in single subject data the false positive rates were inversely correlated with the amount of spatial smoothing^69^, so that a smaller Gaussian kernel might have produced invalid results. For LISA, we did not apply any spatial smoothing. The results are shown in Figs. 5, 6 and Supplementary Figures 13–16. Note that the maps derived by LISA follow the expected order of finger representations in primary somatosensory cortex but are spatially more specific than the smoothed maps obtained by SPM.

The other two methods (FSL-TFCE, AFNI) are not directly applicable because they use permutation of volumes to derive the null distribution. This is not a problem in group-level studies where each volume corresponds to one subject so that individual volumes can be regarded as exchangeable. However, in the case of a single subject analysis, volumes correspond to time steps that are not exchangeable because of temporal autocorrelations. In LISA, this problem does not arise because we shuffle task labels rather than volumes. However, to still allow for some form of comparison, we cut the data into small chunks corresponding to the 26 individual trials with a duration of 9 s each. We then obtained a contrast map for each trial and performed an analysis using AFNI and FSL assuming that trials are exchangeable, see Supplementary Figure 15. Note that this approach is not feasible for event-related designs.

### Simulations for checking false positive rates at 3 Tesla

To make sure that LISA does not produce inflated false positive rates, we subjected it to the test described by Eklund et al.^8^. We found that the false positive rates were well within the acceptable range of 5%. For details, see Supplementary Methods 1, Supplementary Figure 1, and Supplementary Table 1.

### Simulations for checking false positive rates at 7 Tesla

To check whether LISA produces inflated false positive rates when applied to single subject data at ultrahigh fields we modified the tests proposed by Eklund et al.^8,69^. Specifically, we used resting state 7 T fMRI data of the HCP of 25 subjects^60,62^, and applied a variety of “fake” experimental designs. We found that the false positive rates were well below the acceptable level of 5%. For details, see Supplementary Methods 2, Supplementary Figure 2, and Supplementary Table 2.

### Simulations for power calculations

In addition, we performed a range of simulations for power calculations. The details are described in Supplementary Methods 5, Supplementary Table 3, and Supplementary Figures 26–32.

In brief, we first generated background noise using a non-Gaussian autocorrelation function using the model proposed by Cox et al.^70^ with various parameters settings, see Supplementary Figure 27. We then added synthetic signals using signal-to-noise ratios in the range of [0.4, 1.0]. The signals had various shapes and sizes. We have used stick-like shapes to test the sensitivity to detect fine-grained structures in ultra-high resolution images. We also used spheres of various sizes to test the sensitivity w.r.t. area size, see Supplementary Figure 28. For each simulation run, we generated 26 maps to simulate a typical sample size of fMRI group studies. Finally, we performed statistical inferences using the same four methods as in Figs. 2, 3 and calculated their power. The results are shown in Supplementary Figures 29–32. We found that all methods performed similarly well in detecting signals that are large and strong. However, LISA was clearly superior in detecting signals that are small or medium in size. This agrees with the results shown in Fig. 3.

## Discussion

Here, we have introduced the new method LISA for statistical inference of fMRI data that receives a power boost from a nonlinear edge-preserving filter. In our experiments, we found that LISA was considerably more sensitive compared to other methods and required much smaller samples to achieve similar results. Considering the cost of data acquisition, this has a direct impact on the possibility of detecting true activations. Importantly, the increase in sensitivity was achieved without inflating false positive rates and without compromising spatial specificity.

This latter point is particularly relevant as it makes LISA suitable for high-resolution imaging. Ultrahigh field MRI (≥7 Tesla) is a rapidly growing field that for the first time allows investigating structure-function correspondences at the meso- and micro-scale in the living human brain. Spatial precision is crucial in this context^34,71–73^.

We found that small activations are in the blind spot of cluster-based methods. Using a CDT of *p* < 0.01, AFNI could only detect regions that were larger than the entire thalamus. At a more stringent threshold, smaller regions were detected, however at the cost of a severe loss in sensitivity. Similar observations hold for SPM. The threshold-free algorithm FSL-TFCE avoids the problem of defining a CDT. However, its spatial specificity is also poor as can be seen from the high variability in the sizes of the smallest clusters it finds (Fig. 3), see also ref. ^27^. We therefore conclude that voxel-level methods such as the one proposed here offer clear advantages over cluster-based methods.

Importantly, the boost in statistical power achieved by LISA is not merely due to its use of FDR which by definition is less conservative than FWER. Rather, it is the spatially adaptive filter that is crucial in securing LISA’s advantage. This became evident from a direct comparison of results with and without the bilateral filter, see Supplementary Figs. 23, 24. Furthermore, we found virtually no difference between the FDR and FWER results obtained using SPM. This suggests that the initial CDT largely determines the final result rather than the type of error that is controlled, see also ref. ^74^.

In conclusion, we hope that because of its improved sensitivity and better spatial specificity, LISA will help in developing novel and more realistic models of human brain function.

## Software availabilty

The LISA software is available at https://github.com/lipsia-fmri/lipsia. The other software packages (SPM, AFNI, FSL) are available from their respective websites.

## Electronic supplementary material

Supplementary Information

## Data Availability

Data of the Human Connectome Project are available from www.humanconnectomeproject.org (1200 Subjects Data Release, Release Date: March 01, 2017). Resting state data used for the “Eklund test” (“Beijing sample”) are available as described in ref. ^8^. The fMRI data acquired of a single subject at 7 Tesla are available upon request from the corresponding author.
