# Supplementary material for: LISA improves statistical analysis for fMRI
Source: Nat Commun. 2018 Oct 1;9:4014. doi: 10.1038/s41467-018-06304-z (PMC6167367; doi:10.1038/s41467-018-06304-z)
Supplement: Supplementary file 1 — Supplementary Information [file 41467_2018_6304_MOESM1_ESM.pdf]

Supplementary Information

LISA improves statistical analysis for fMRI

Lohmann et al.

Supplementary Note 1: Pseudocode LISA, onesample test

Input: contrast images $A_1, ..., A_n$, number of permutations K, FDR threshold α
Output: Activation map M corrected for FDR

```
function LISA(A_1, ..., A_n, K, α)
    B_z, H_orig ← GetHist(A_1, ..., A_n)

    for p ∈ {1, ..., K} do                              ▷ Random permutations
        H_p ← GetHist(RandomSignSwitch(A_1, A_2, ..., A_n))
    end for

    Fdr ← GetFdr(H_orig, H_1, ..., H_K)
    M ← Voxelwise threshold applied to image B_z such that Fdr < α
    (return M)

end function


function GetHist(A_1, ..., A_n)
    Z ← Ttest(A_1, ..., A_n)                            ▷ Voxelwise t-test
    B ← Bilateralfilter(Z)                              ▷ Bilateralfilter applied to Z
    H ← Histogram(B)                                    ▷ Histogram H of image B
    return(H, B)
end function


function GetFdr(H_z, H_1, ...H_K)
    F_z ← CDF(H_z)                                      ▷ Cumulative distribution function of non-permuted
                                                          histogram
    H_0 ← Sum(H_1, ...H_K)                              ▷ Sum of all null histograms
    F_0 ← CDF(H_0)                                      ▷ CDF of merged null histograms
    Fdr(x) ← F_0(x)/F_z(x)                              ▷ False discovery rate function
    return(Fdr)

end function


procedure RandomSignSwitch(A_1, ..., A_n)

    for p ∈ {1, ..., n} do
        with probability 0.5 switch signs of all voxel values in A_i
    end for
end procedure
```

Supplementary Methods 1: False positive rates in group level 3 Tesla data

To make sure that LISA does not produce inflated false positive rates, we subjected it to the test described by Eklund et al. [1]. We used the same four experimental designs (B1,B2,E1,E2) and the same 198 resting state data sets of the Beijing sample as in [1], as well as the same preprocessing pipeline which consisted of a correction for motion, an alignment with the MNI template, and four different levels of spatial smoothing. After preprocessing, we used SPM [2] to compute GLM-based contrast maps for each subject, each of the four "fake" designs and the four levels of spatial smoothing resulting in a total of 4x4x198=3168 contrast maps. We then randomly drew 1000 samples of size twenty from each of the 4x4 different sets of maps, and applied a group-level onesample t-test using LISA on each of these samples. By definition, every activation found in this scenario is a false positive. We found that the false positive rates were well within the acceptable range of 5%, see supplementary table S1 below.

	$(2mm)^3$ resolution				$(3mm)^3$ resolution			
	B1	B2	E1	E2	B1	B2	E1	E2
4mm	0.042	0.025	0.042	0.014	0.047	0.017	0.028	0.007
6mm	0.039	0.014	0.030	0.013	0.049	0.025	0.031	0.007
8mm	0.041	0.018	0.029	0.015	0.053	0.018	0.027	0.018
10mm	0.045	0.023	0.023	0.007	0.040	0.022	0.038	0.008

Supplementary Table 1: Results of the Eklund test applied to 3 Tesla group level data. The numbers denote the percentage of tests in which at least one voxel was a false positive. The tests were performed using two different preprocessing regimes in which the data were either resampled to $(2mm)^3$ or to $(3mm)^3$ resolution.

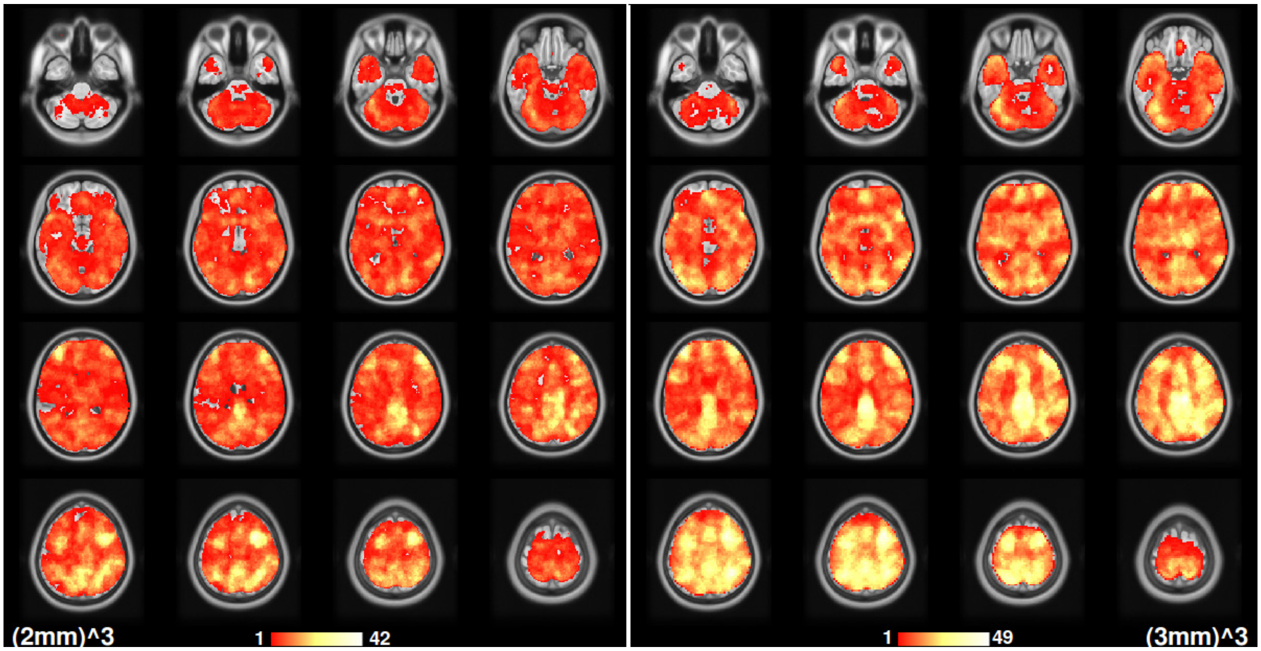

Supplementary Figure 1: Distribution of false positives of table 1. This figure shows the spatial distribution of the false positives across all experimental designs and all test subjects totalling 8×1000 data sets for the $(2mm)^3$ (left) and the $(3mm)^3$ preprocessing regime (right). The color code indicates the total number of false positives per voxel. In the $(2mm)^3$ preprocessing regime, the largest number of false positives per voxel is 42 out of 8000. In the $(3mm)^3$ preprocessing regime, it is 49.

Supplementary Methods 2: False positive rates in single subject 7 Tesla data

To check whether LISA produces inflated false positive rates when applied to single subject data at ultrahigh fields we modified the tests proposed by Eklund et al. [1, 3]. Specifically, we used resting state 7T fMRI data of the Human Connectome Project (HCP) of 25 subjects [4, 5]. All data sets were acquired with the following parameters: TR=1000ms, 1.6 mm isotropic voxel size. Data were acquired in four runs. Here we only used the first two runs. Each run had a duration of 15 minutes and contained 900 volumes. The preprocessing protocol is described in [6].[1] Artefacts due to motion, scanner noise, and other nuisance sources were removed using FSL-Fix [8, 9]. No spatial smoothing was applied.

We randomly generated 40 different experimental "fake" designs of four types each, namely two types of block designs (B1, B2) and two types of event-related designs (E1, E2). All designs simulated two different experimental conditions separated by intertrial "resting" periods. The designs of type B1 had trials of length 10 seconds, and the B2 designs had trials of length 20 seconds. The intertrial duration was 6 seconds. The event-related designs E1, E2 had variable intertrial durations with a mean duration of 6 seconds and standard deviation of 2 seconds. The trials of the E1 design had a duration of 2 seconds, those of E2 were 1 second long. We applied the canonical hemodynamic response function [10] and computed the contrast between the two experimental conditions. We performed 4x40 tests for each subject so that for each type of design we had a total of 1000 tests per condition. We performed two sets of tests using either one run with a duration of 15 minutes, or two runs with a total duration of 30 minutes. If two runs were used, to ensure exchangeability random permutations of task labels were only done within the same run. We recorded the false positive rate, i.e. the number of cases in which at least one voxel passed the significance threshold, see table 2 below.

[1] Recently, a problem in the preprocessing pipeline related to the geometric unwarping of 7T HCP data was reported [7]. In the present context, we use these data only as a null model for statistical inference where geometric accuracy is irrelevant. Therefore, we have decided to continue to use these data in spite of this problem.

	one run				two runs			
subject ID	B1	B2	E1	E2	B1	B2	E1	E2
100610	0	3	0	0	0	2	1	1
102311	1	0	1	0	0	2	0	1
104416	2	2	0	2	0	0	0	0
105923	2	7	1	1	1	2	0	0
111312	2	1	1	0	0	2	0	0
111514	0	1	1	2	1	1	0	0
114823	0	1	0	1	2	1	1	0
115017	0	0	1	1	0	0	1	2
118225	0	1	1	0	1	1	1	0
125525	1	0	0	0	1	2	0	1
128935	1	0	0	1	0	2	1	0
131722	0	1	2	0	0	0	1	1
137128	0	2	0	0	3	2	0	0
140117	3	2	0	0	1	3	0	1
144226	1	1	1	1	0	0	0	1
146129	1	1	0	1	2	2	1	0
146432	1	0	1	0	1	0	0	1
150423	1	2	1	1	1	0	1	1
155938	1	2	2	1	0	1	0	1
156334	1	0	1	0	2	1	0	0
157336	1	0	1	0	3	1	0	1
158035	1	1	0	0	1	1	0	1
158136	2	2	0	2	2	1	2	0
162935	2	3	3	0	0	2	1	1
164131	0	0	1	1	0	1	0	0
False pos rate	0.024	0.033	0.019	0.015	0.022	0.030	0.011	0.014

Supplementary Table 2: Results of the Eklund test applied to 7 Tesla single subject data. The numbers denote the number of tests in which at least one voxel was a false positive. For example, in subject 100610 (one run), three of the 40 tests using the design type B2 produced such an error. The sum of false positives over all subjects and design type B2 is 33, and the corresponding false positive rate is 33/1000 = 0.033.

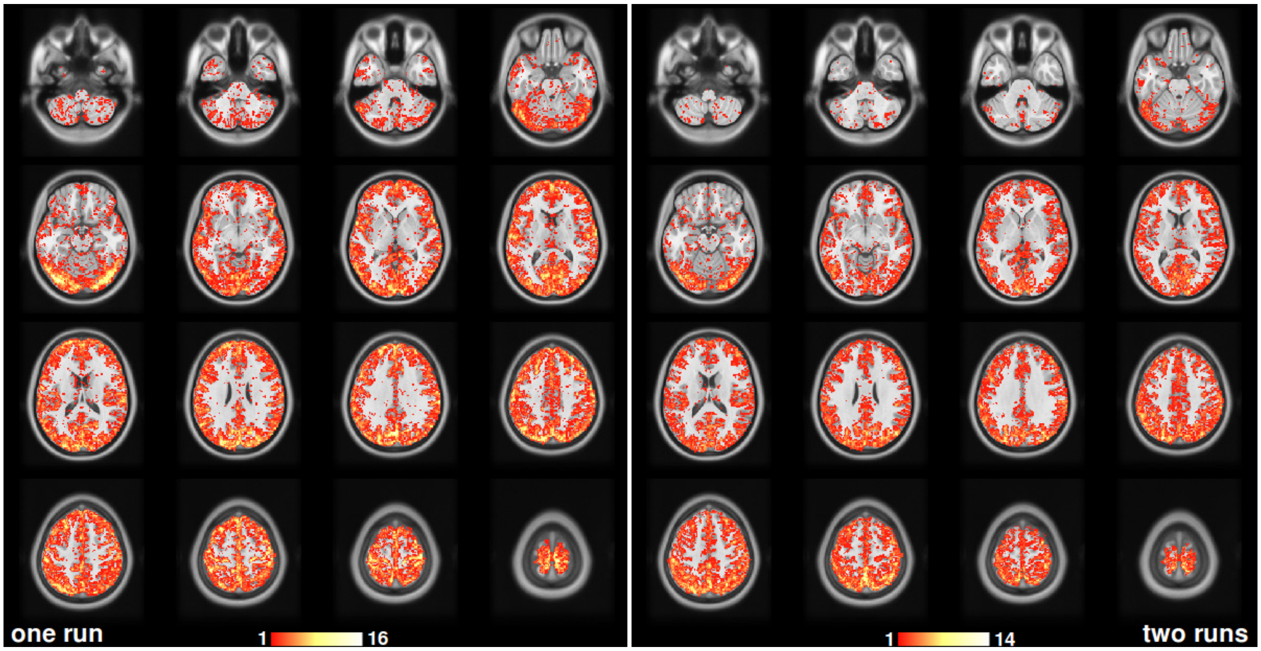

Supplementary Figure 2: Distribution of false positives of table 2. This figure shows the spatial distribution of the false positives across all experimental designs and all test subjects using either one run (left) or two runs (right). The color code indicates the total number of false positives per voxel.

Supplementary Methods 3: Human Connectome Project data (3 Tesla)

Experimental design. In the motor task, participants were cued visually to tap their left or right fingers, squeeze their left or right toes, or move their tongue. Each block lasted 12 seconds (10 movements), and was preceded by a 3 second cue. Here we investigated only the left hand fingertapping condition. In the emotion experiment, participants were cued to decide which of two faces matched the face shown on top of the screen. In a second experimental condition, an analogous task was done using shapes instead of faces. The faces had either angry or fearful expressions. Here we investigated the contrast "faces minus shapes". For further details see [11].

Imaging parameters. We used data of the left-right phase encoding runs. All data sets were acquired with the following parameters: TR=720ms, TE=33.1ms, 2 mm isotropic voxel size, multiband factor 8. The preprocessing protocol is described in [6]. In addition, FSL-Fix [12–14] was used for data denoising, removing physiological nuisance effects as well as motion and multi-band artifacts. To account for inter-subject variations, we applied a Gaussian spatial filter with a kernel size of 6 mm. Using FSL-FEAT [12–14], we performed individual one-sample t-tests resulting in 400 single-subject input maps of contrast values.

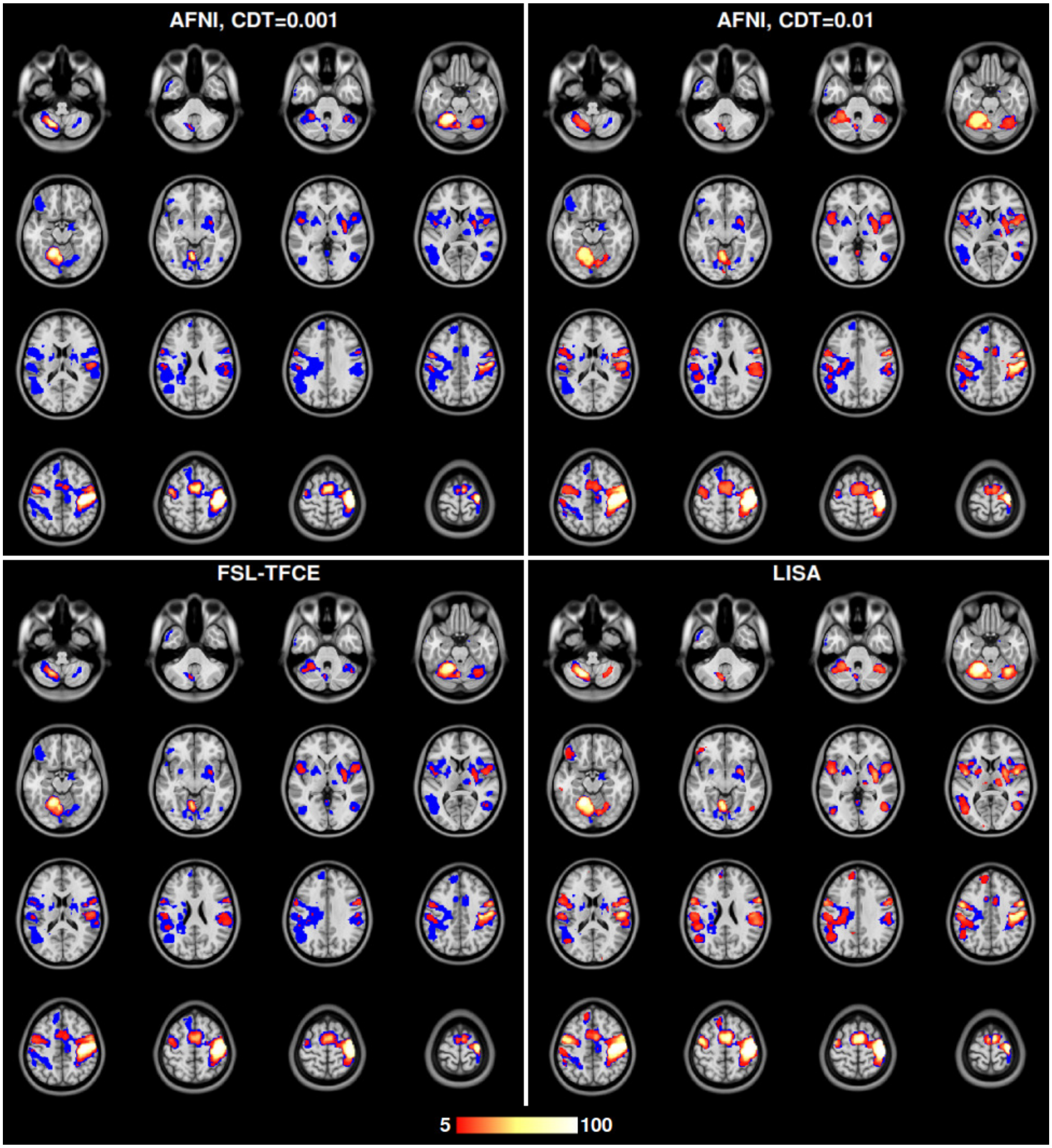

Supplementary Figure 3: Reproducibility maps, motor task, (AFNI, FSL-TFCE, LISA). The colors represent reproducibility scores, see Fig. 2 of the main text. The top images show results obtained using AFNI with two different initial cluster defining thresholds CDT=0.001 and CDT=0.01. The bottom images show results obtained using FSL-TFCE and LISA. The underlying blue areas show the reference map which was derived using all 400 subjects.

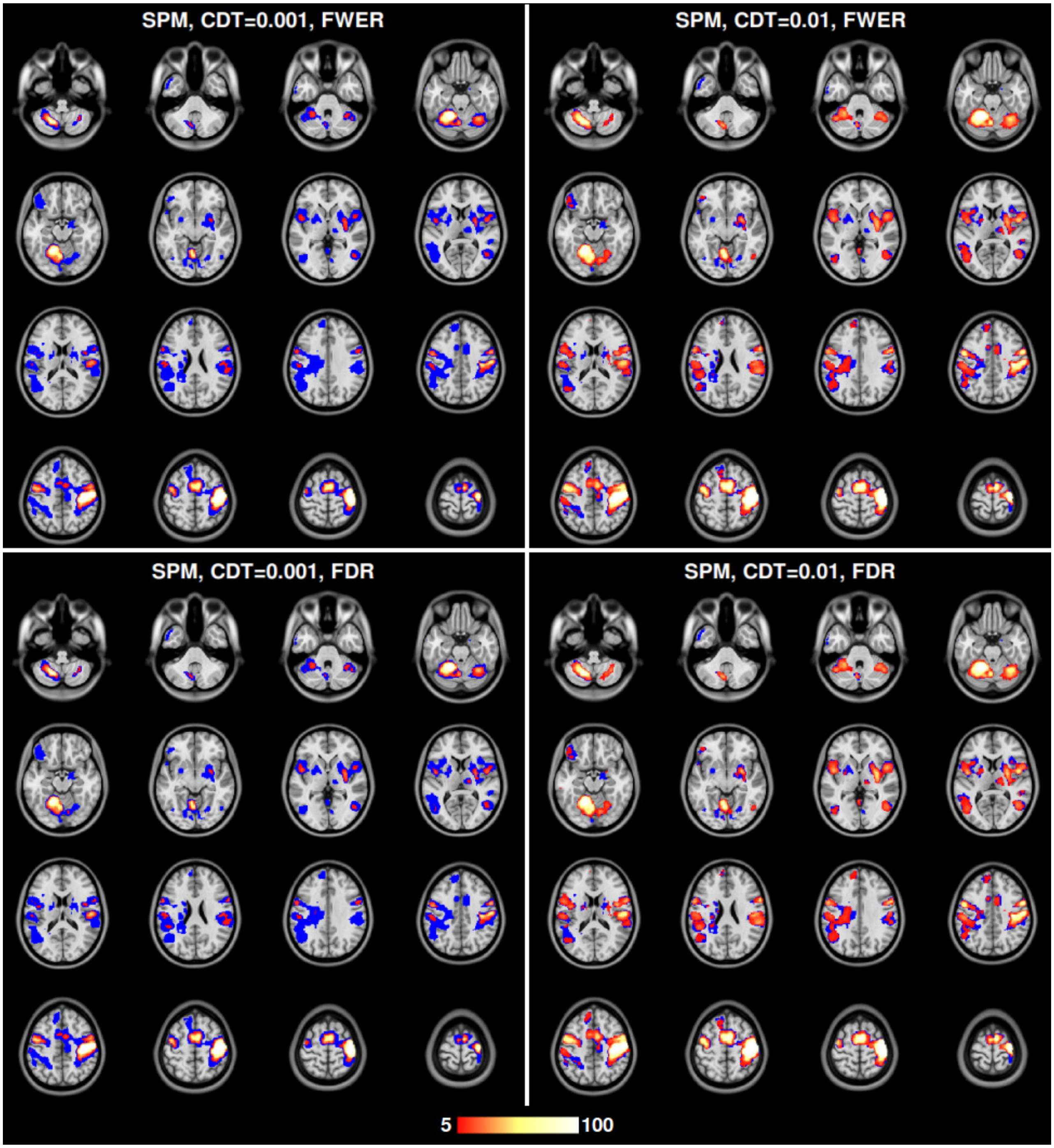

Supplementary Figure 4: Reproducibility maps, motor task, (SPM). The colors represent reproducibility scores, see Fig. 2 of the main text. The images show results obtained using SPM with two different initial cluster defining thresholds CDT=0.001 and CDT=0.01, and corrected for the familywise error (top) and the false discovery rate (bottom). Note that the FDR results are almost identical to FWER results.

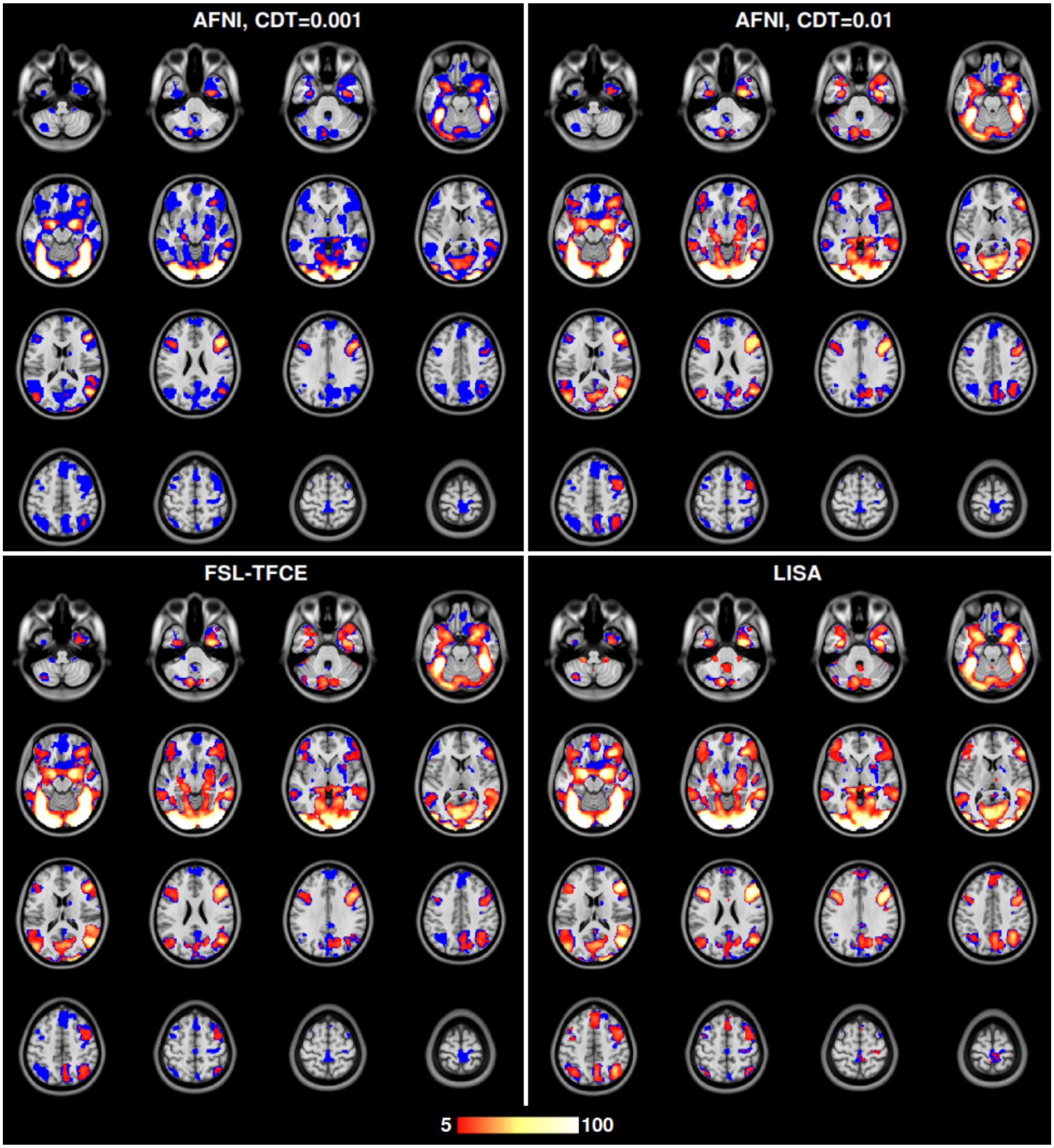

Supplementary Figure 5: Reproducibility maps, emotion task, (AFNI, FSL-TFCE, LISA). The colors represent reproducibility scores, see Fig. 2 of the main text. The top images show results obtained using AFNI with two different initial cluster defining thresholds CDT=0.001 and CDT=0.01. The bottom images show results obtained using FSL-TFCE and LISA. The underlying blue areas show the reference map which was derived using all 400 subjects.

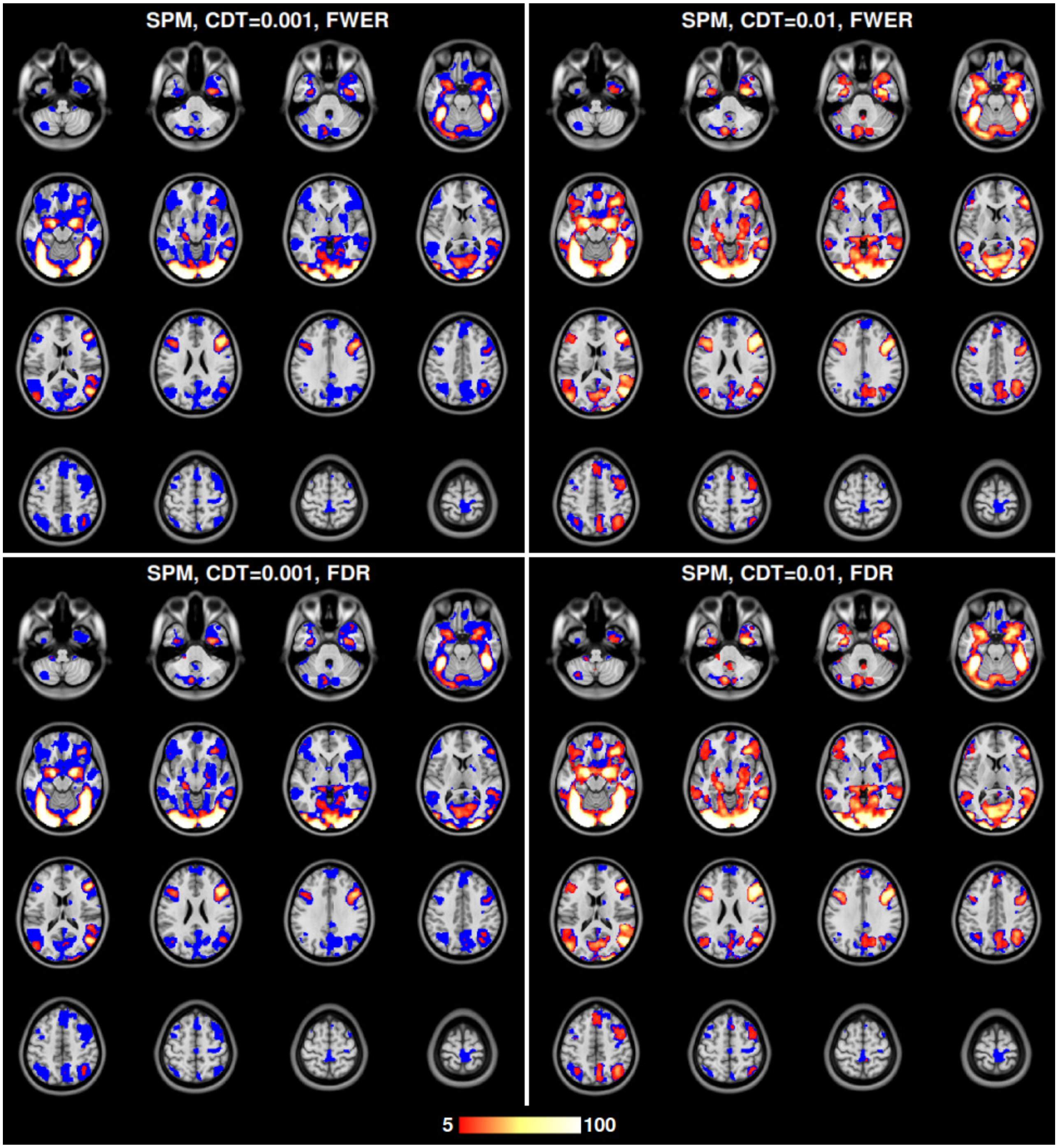

Supplementary Figure 6: Reproducibility maps, emotion task, (SPM). The colors represent reproducibility scores, see Fig. 2 of the main text. The images show results obtained using SPM with two different initial cluster defining thresholds CDT=0.001 and CDT=0.01, and corrected for the familywise error (top) and the false discovery rate (bottom). Note that the FDR results are almost identical to FWER results.

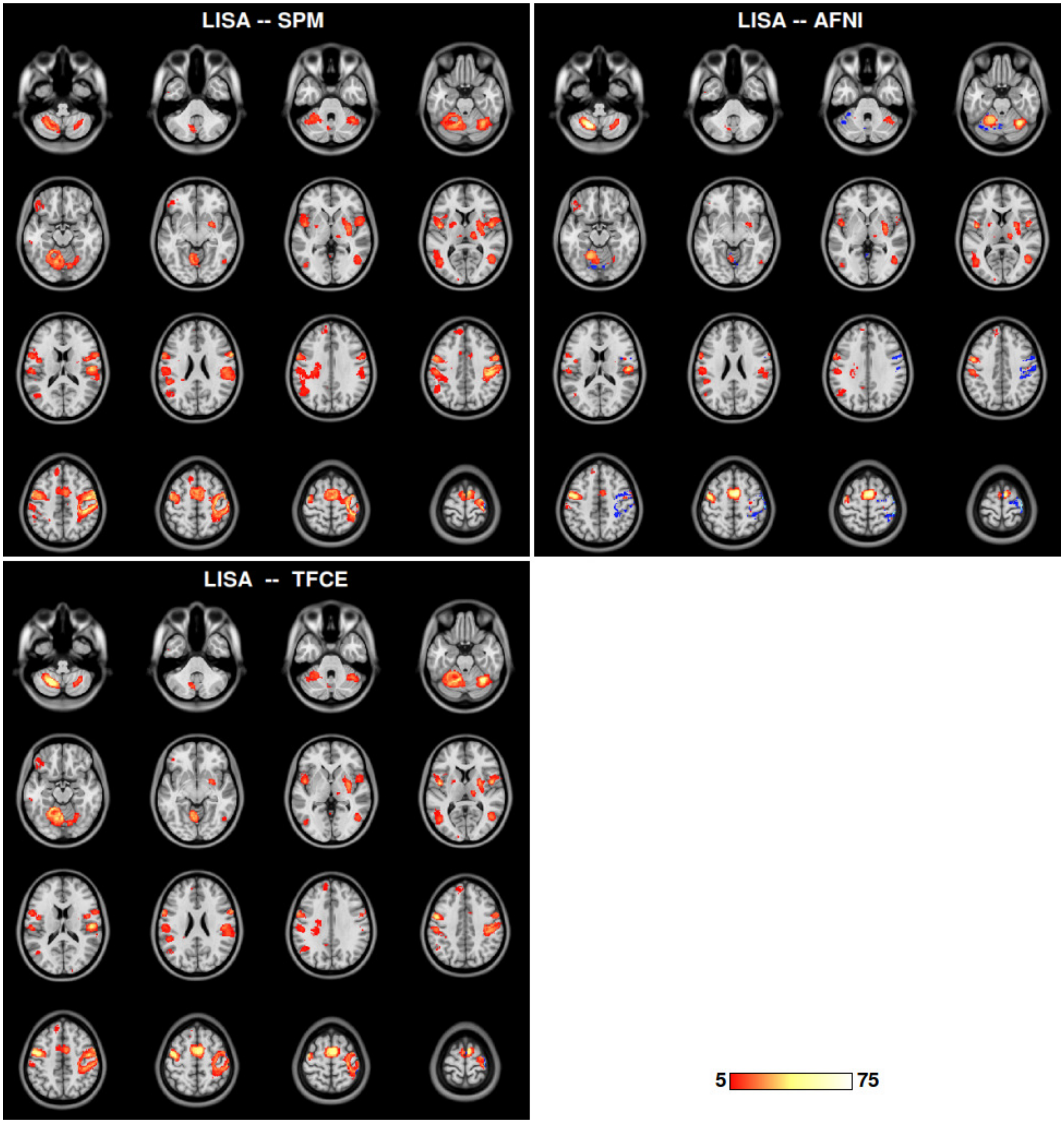

Supplementary Figure 7: Differences of reproducibility maps (motor task). The maps show a voxelwise subtraction of reproducibility maps (Supplementary Figures 3,4). The three difference maps show LISA - AFNI (CDT=0.01), LISA - SPM (CDT=0.001), and LISA - FSL.

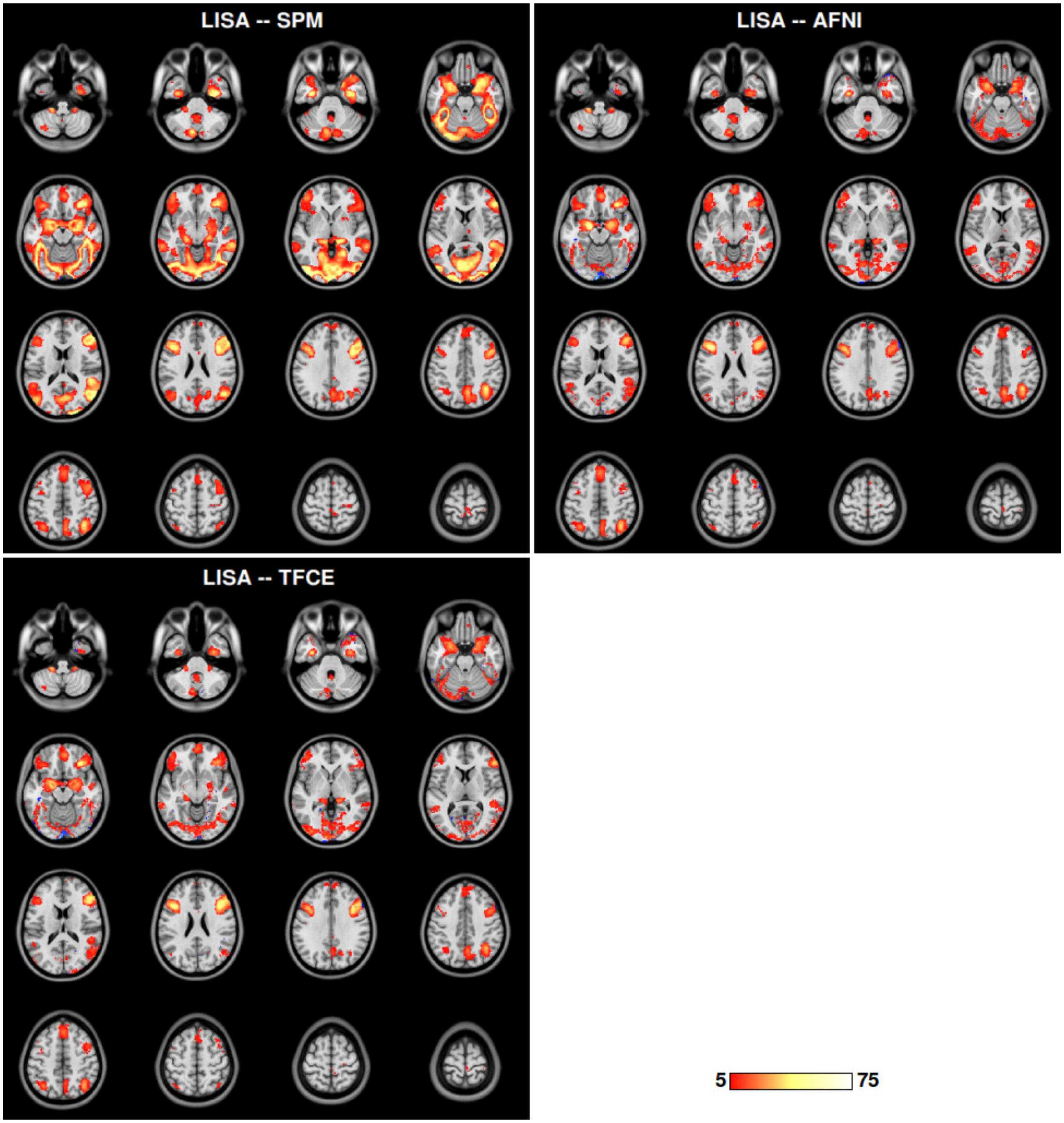

Supplementary Figure 8: Differences of reproducibility maps (emotion task). The maps show a voxelwise subtraction of reproducibility maps (Supplementary Figures 5,6). The three difference maps show LISA - AFNI (CDT=0.01), LISA - SPM (CDT=0.001), and LISA - FSL.

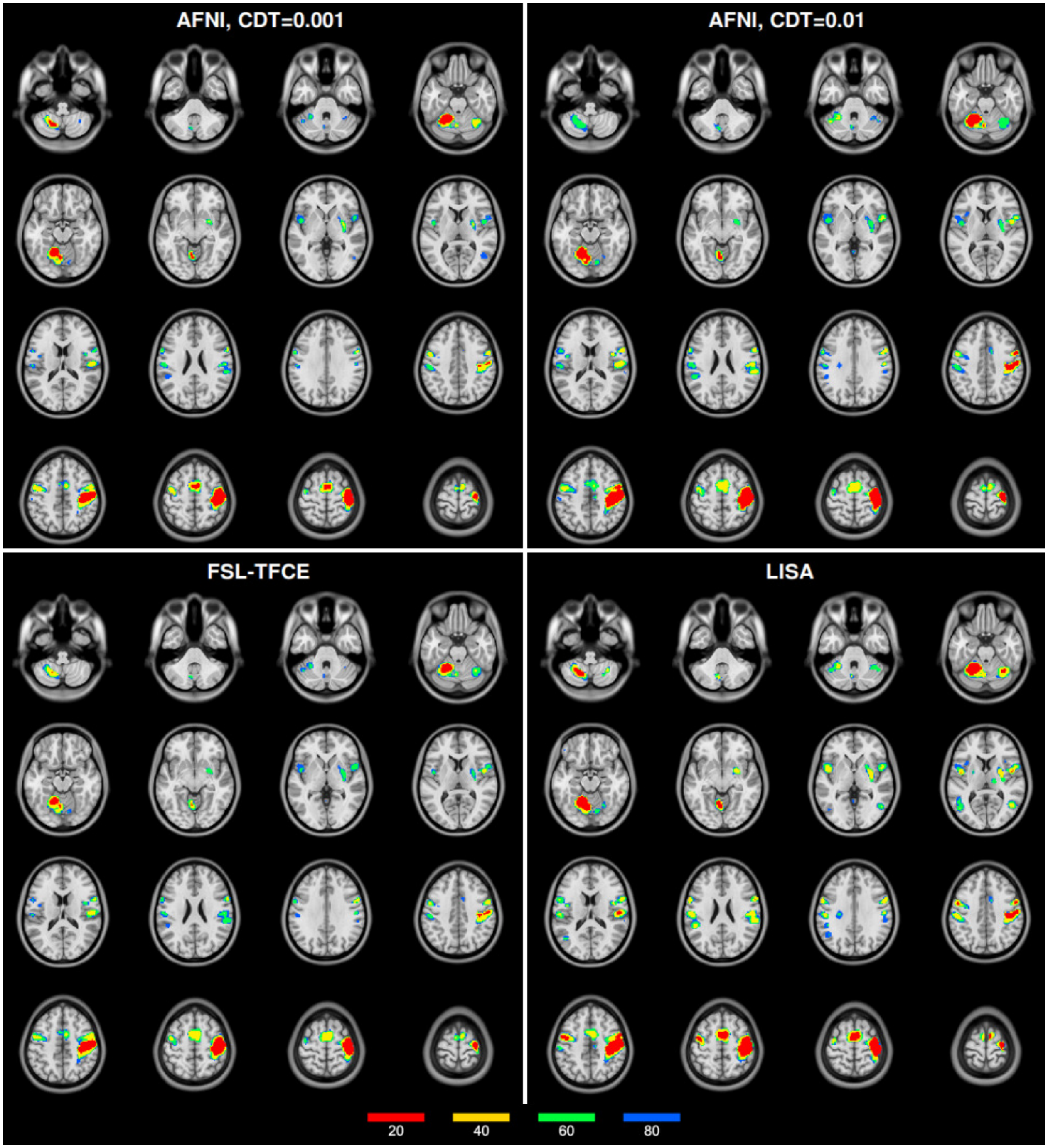

Supplementary Figure 9: Sample size maps, Motor task, (AFNI, TFCE, LISA). The colors represent minimal sample sizes, see Fig. 3 of the main text. The top images show results obtained using AFNI with two different initial cluster defining thresholds CDT=0.001 and CDT=0.01. The bottom images show results obtained using TFCE and LISA.

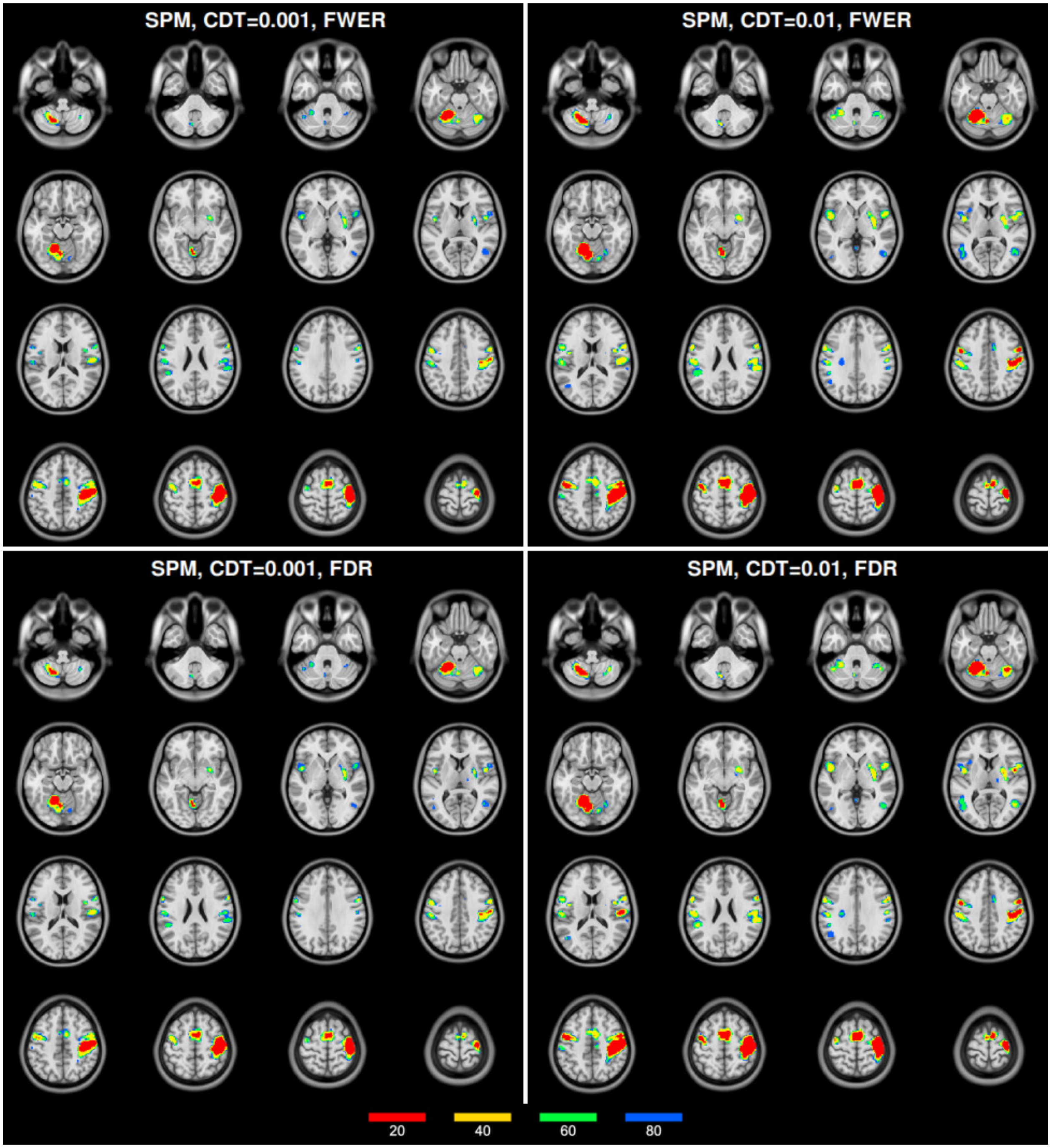

Supplementary Figure 10: Sample size maps, Motor task, (SPM). The colors represent minimal sample sizes, see Fig. 3 of the main text. The images show results obtained using SPM with two different initial cluster defining thresholds CDT=0.001 and CDT=0.01, and corrected for the familywise error (top) and the false discovery rate (bottom). Note that the FDR results are almost identical to FWER results.

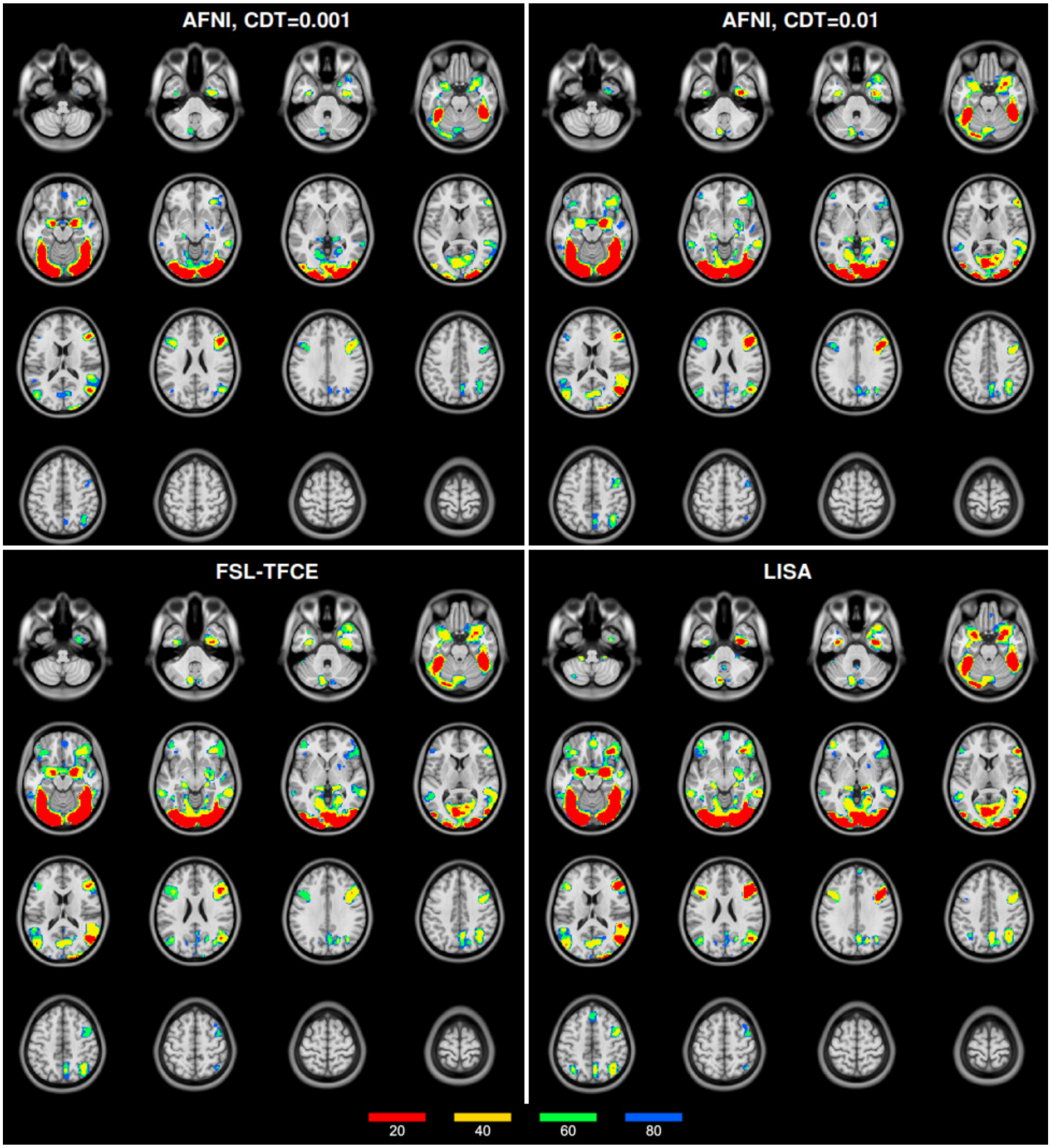

Supplementary Figure 11: Sample size maps, Emotion task, (AFNI, TFCE, LISA). The colors represent minimal sample sizes, see Fig. 3 of the main text. The top images show results obtained using AFNI with two different initial cluster defining thresholds CDT=0.001 and CDT=0.01. The bottom images show results obtained using TFCE and LISA.

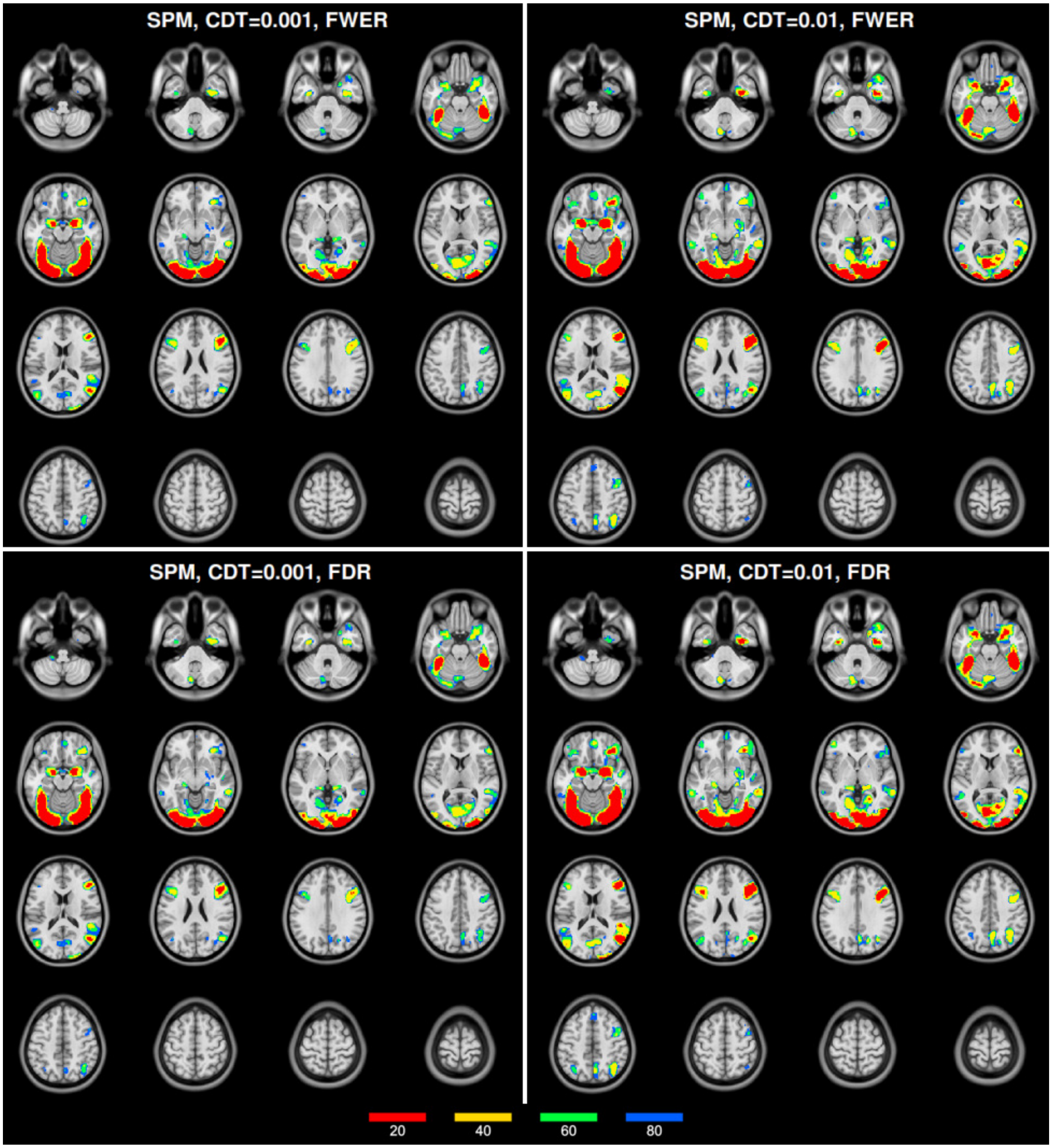

Supplementary Figure 12: Sample size maps, Emotion task, (SPM). The colors represent minimal sample sizes, see Fig. 3 of the main text. The results are obtained using SPM with two different initial cluster defining thresholds CDT=0.001 and CDT=0.01, and corrected for the familywise error (top) and the false discovery rate (bottom).

Supplementary Methods 4: Finger maps at 7 Tesla (single subject data)

Experimental design. A healthy subject (female, aged 25 years) was stimulated in the MR scanner at four fingers of her right hand using an MR-compatible tactile stimulator. The stimulator consisted of four stimulation belts, which stimulated one finger pad at a time. Hand and fingers did not move during stimulation. Surfaces of six different roughness levels were mounted on the belts. During stimulation, the belts moved and thus stimulated the finger pads that rested passively on the belts. After each stimulation, the subject was asked to make a roughness judgment (choosing between level 1=smooth to 6=very rough) about the previous stimulation. Each finger stimulation lasted for 9 seconds (blocked design), and was repeated 13 times per run resulting in 52 trials per run. The stimulation consisted of two runs that were separated by a short break, one forward scan (stimulating first the index finger, followed by the middle finger, ring finger, small finger, back to index, and so forth), and a reverse scan (stimulating first the small finger, followed by the ring finger, the middle finger, the index finger, and so forth). At the start and end of each run, a 15 second fixation screen was presented. Functional imaging data were used to calculate the representation of the fingers in contralateral primary somatosensory cortex, for details see [15].

Imaging parameters. Task-based fMRI data were acquired of a single subject at a 7 Tesla MRI scanner (Magnetom 7T, Siemens Healthcare Sector, Erlangen, Germany) using an EPI sequence, TR=2000ms, TE=18ms, with a spatial resolution of $(1.5mm)^3$ with 43 axial slices covering primary motor cortex and primary somatosensory cortex bilaterally.

Data analysis. We used a standard preprocessing pipeline which included motion correction and a removal of baseline drifts with a highpass filter (cutoff 1/100 Hz). For the SPM analysis, we applied a spatial Gaussian filter with FWHM=4mm during preprocessing. For the LISA analysis, no spatial smoothing was applied. Four contrasts were computed. In each contrast, one finger was contrasted against the other three fingers.

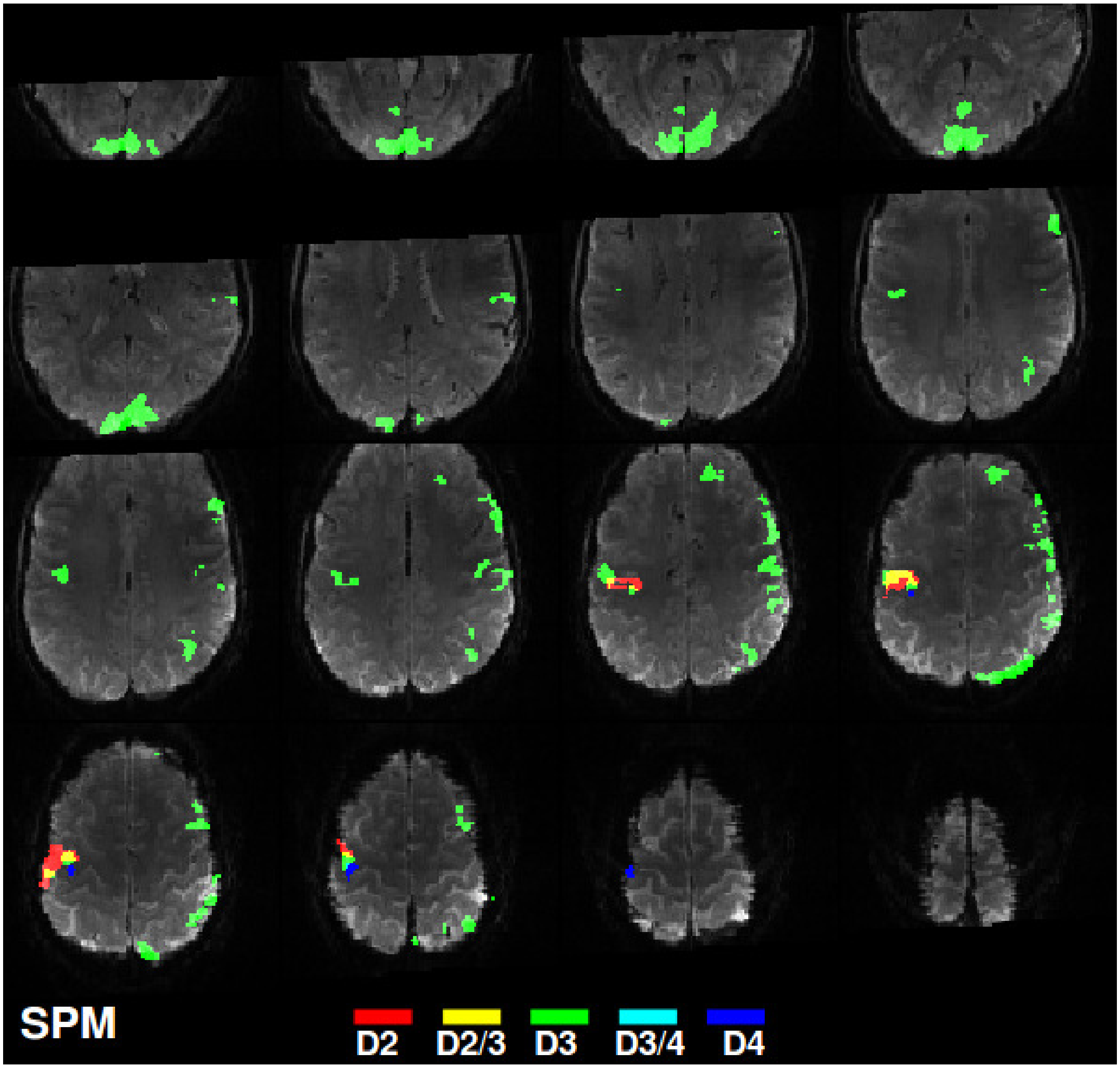

Supplementary Figure 13: Results obtained with SPM (single subject, 7 Tesla fMRI). Four contrasts of a finger stimulation experiment were computed. In each contrast, one finger was contrasted against all other fingers. For clarity, here we only show three of these contrasts. The colors indicate areas that show a statistically significant contrast ($p < 0.05$, corrected) index finger (IF, red), middle finger (MF, green), ring finger (RF, blue). Areas of overlap are marked in yellow (IF+MF) and cyan (MF+RF). Large overlaps indicate a lack of spatial specificity. Note that in the middle finger contrast, SPM discovers several areas outside of the motor area. It is unclear whether these areas are plausible.

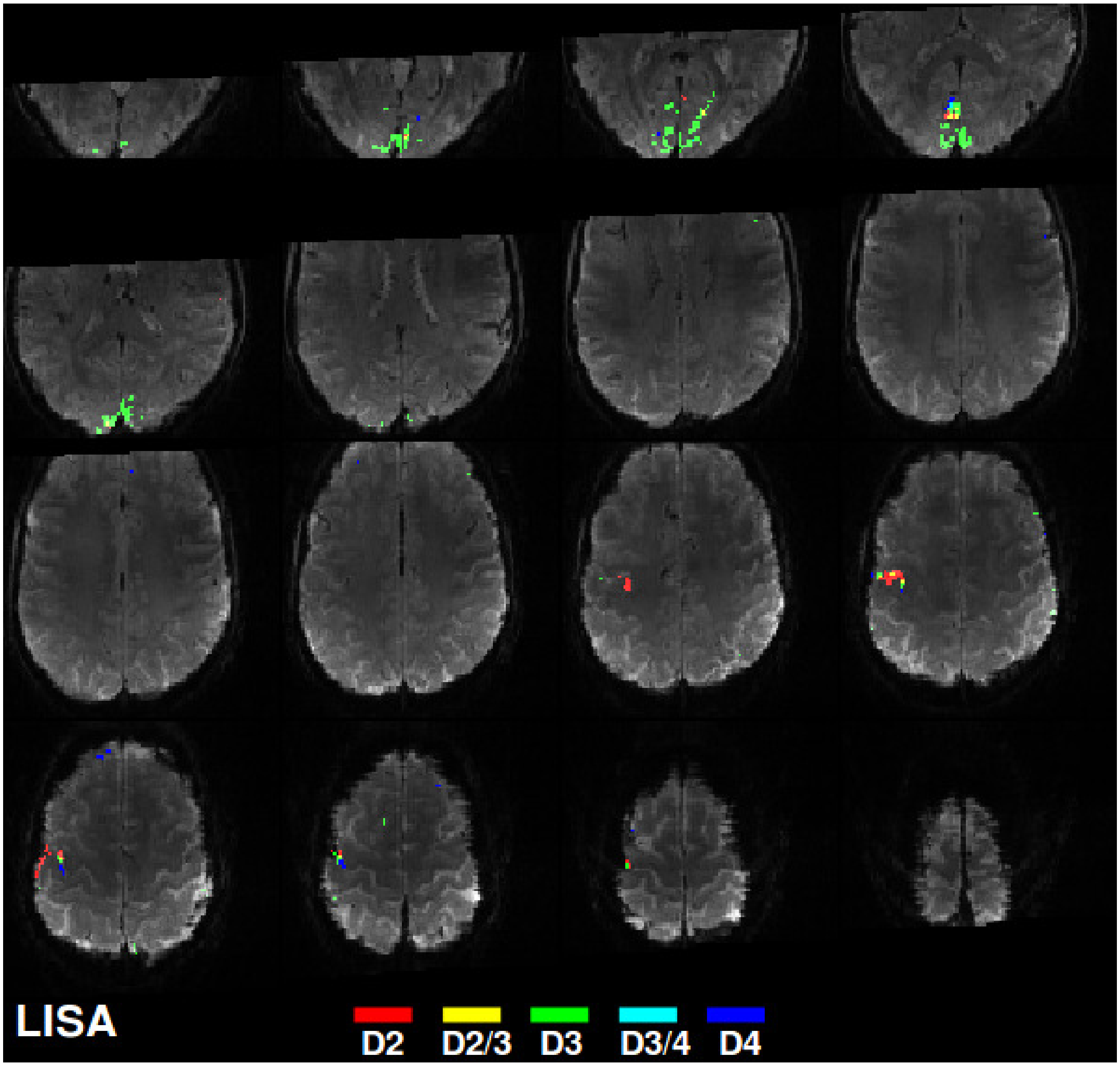

Supplementary Figure 14: Results obtained with LISA (single subject, 7 Tesla fMRI). Four contrasts of a finger stimulation were computed. In each contrast, one finger was contrasted against all other fingers. For clarity, here we only show three of these contrasts. The colors indicate areas that show a statistically significant contrast ($FDR < 0.05$), index finger (IF, red), middle finger (MF, green), ring finger (RF, blue). Areas of overlap are marked in yellow (IF+MF) and cyan (MF+RF). Large overlaps indicate a lack of spatial specificity.

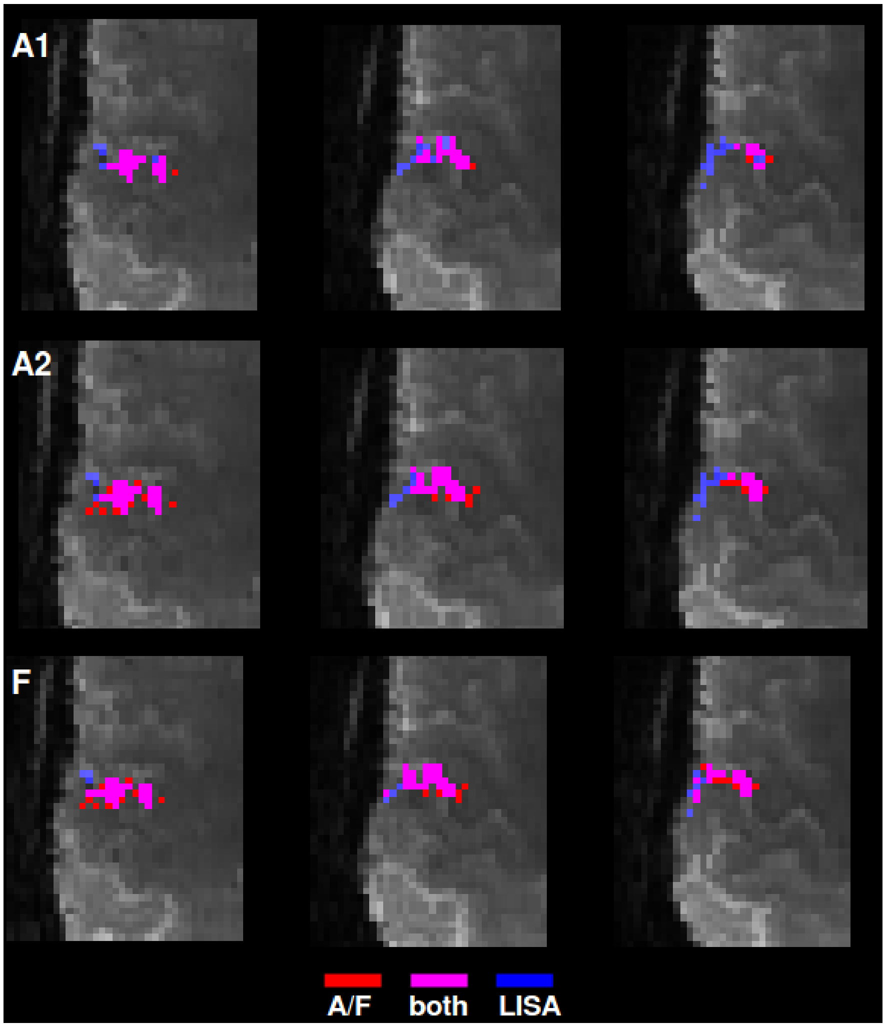

Supplementary Figure 15: Comparison of FSL and AFNI versus LISA (single subject, 7 Tesla fMRI). The figure shows the contrast "index finger versus all other fingers" obtained with AFNI (A1, CDT=0.001), AFNI (A2, CDT=0.01) and FSL-TFCE (F). The results found with AFNI and FSL are shown in red (A/F), the LISA-result is shown in blue. Voxels where the results of LISA and FSL/AFNI agree are shown in magenta (both). The LISA result was obtained by random shuffling of task labels, the results with AFNI and FSL were obtained by cutting the data into small chunks corresponding to the 26 individual trials with a duration of 9 seconds each. This latter approach is only feasible for block designs and assumes that the trials (blocks) are exchangeable. In the motor area, LISA detected 193 voxels. The other results were: AFNI (CDT=0.001) 113 voxels, AFNI (CDT=0.01) 177 voxels, and FSL-TFCE 119 voxels. Thus, LISA was slightly more sensitive.

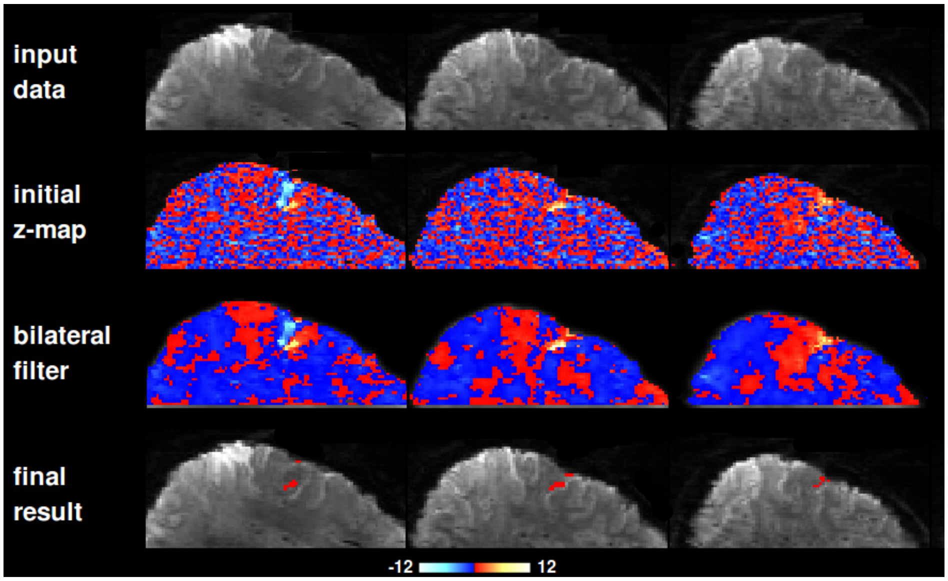

Supplementary Figure 16: Workflow of LISA (stimulation of the index finger, 7 Tesla fMRI). The input into LISA is an unsmoothed fMRI data set. An initial map of z-values uncorrected for multiple comparisons is c omputed. A bilateral filter is applied to this map, and the false discovery rate is computed for every voxel. The final result is a map thresholded such that voxels with Fdr < 0.05 remain.

HCP Motor task

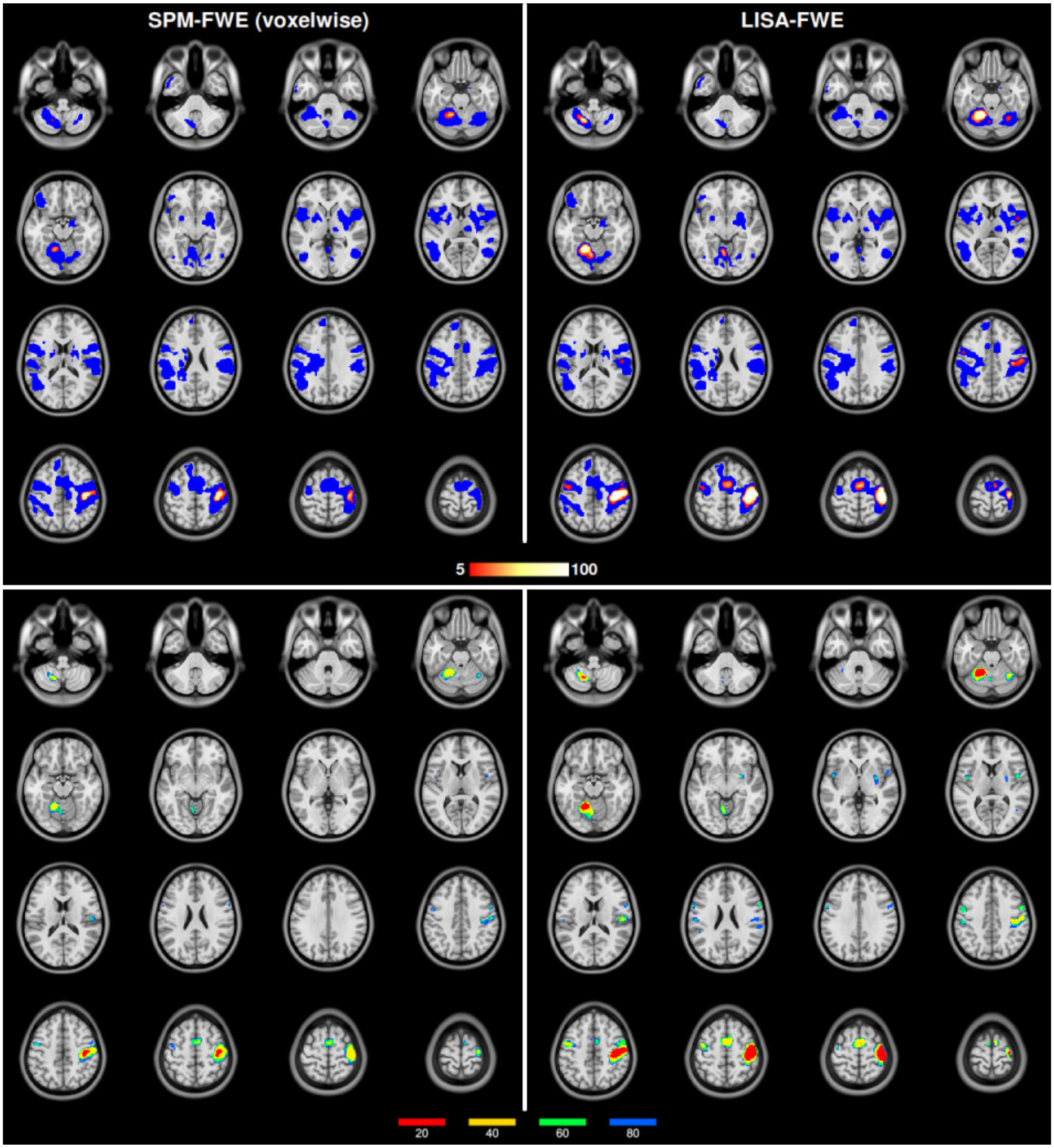

Supplementary Figure 17: Voxelwise familywise error correction (FWE), HCP motor task. The two top images show reproducibility across 100 tests based on randomly drawn samples of size twenty. The colors represent reproducibility scores, i.e. the number of tests in which a given voxel consistently passed the significance threshold. The underlying blue areas show the reference map which was derived using all 400 subjects, see Fig 2 of the manuscript. The two bottom images show minimal sample sizes needed in order to detect an activation with reasonable chance of success, i.e. in at least 50 of 100 tests, see Fig 3 of the manuscript.

HCP Emotion task

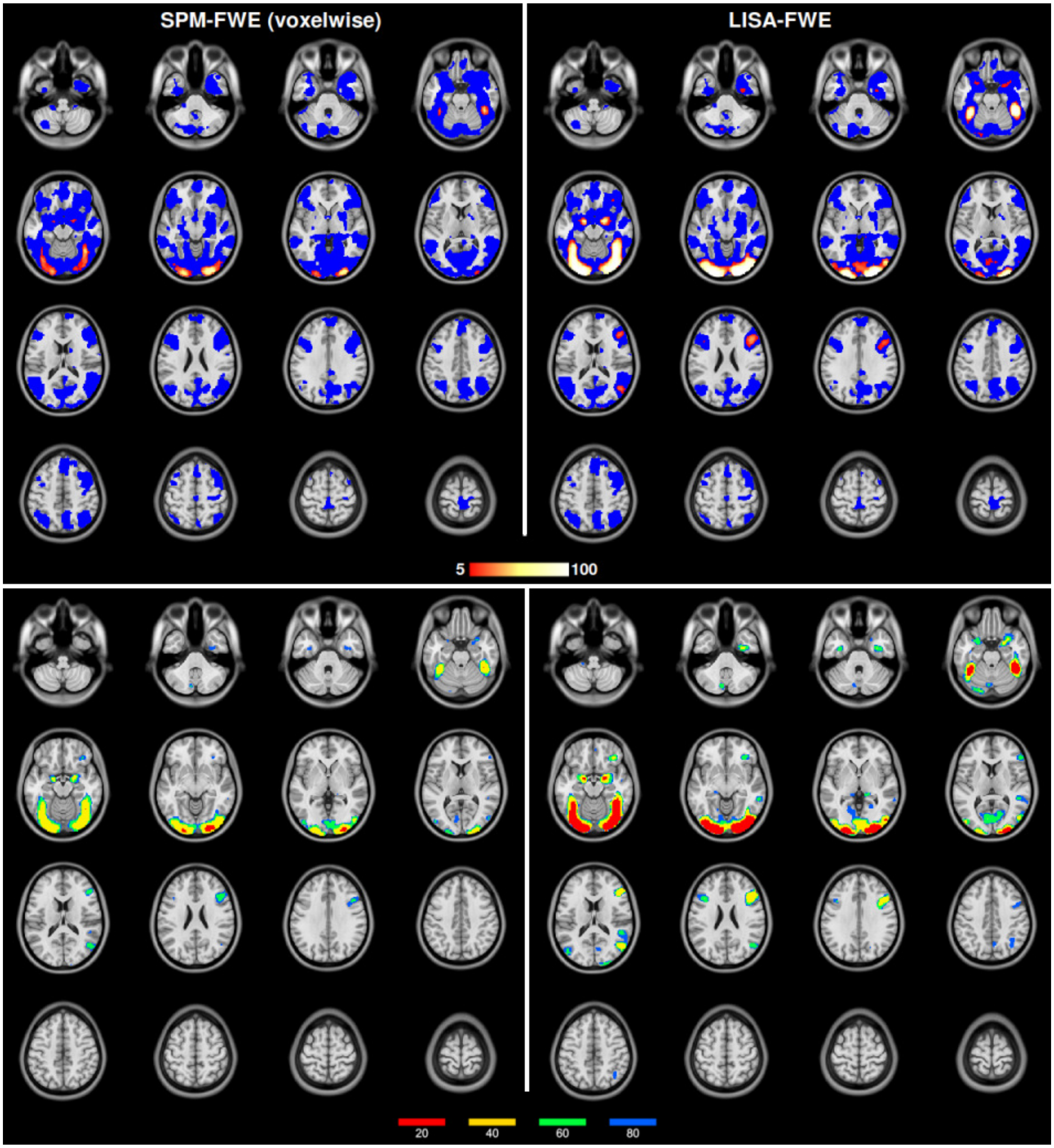

Supplementary Figure 18: Voxelwise familywise error correction (FWE), HCP emotion task. The two top images show reproducibility across 100 tests based on randomly drawn samples of size twenty. The colors represent reproducibility scores, i.e. the number of tests in which a given voxel consistently passed the significance threshold. The underlying blue areas show the reference map which was derived using all 400 subjects, see Fig 2 of the manuscript. The two bottom images show minimal sample sizes needed in order to detect an activation with reasonable chance of success, i.e. in at least 50 of 100 tests, see Fig 3 of the manuscript.

HCP Motor task

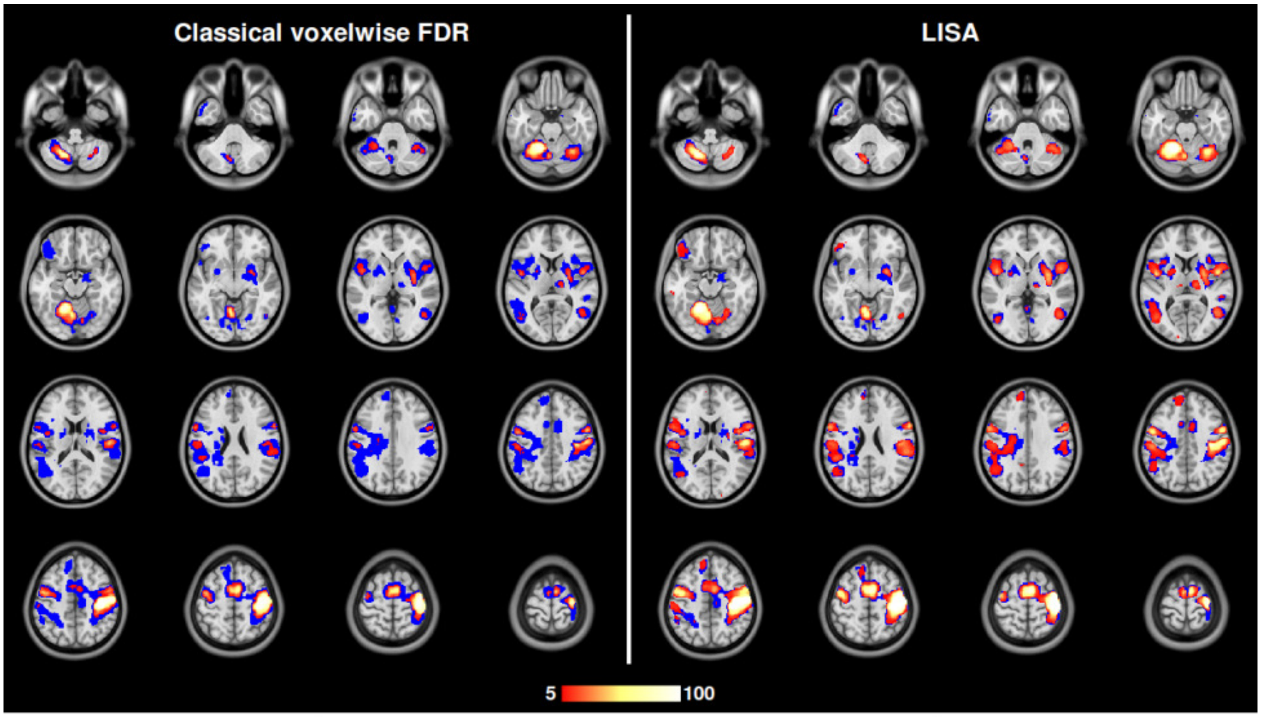

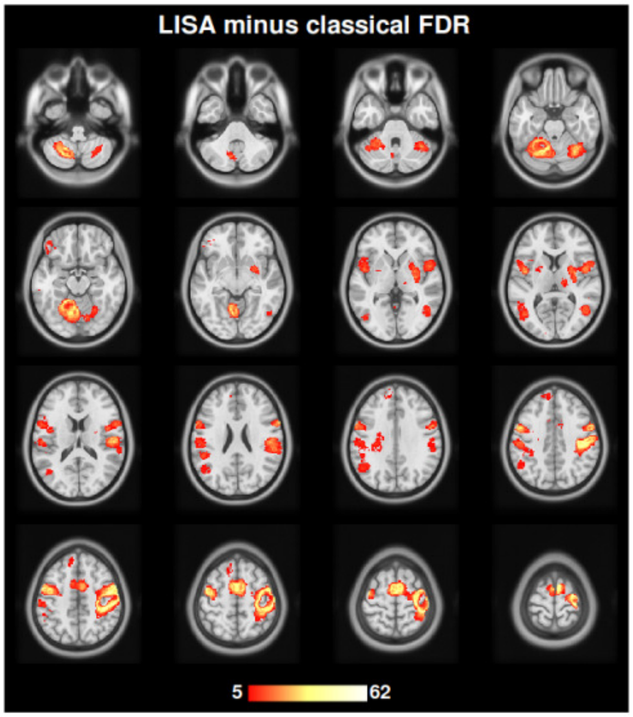

Supplementary Figure 19: Reproducibility maps, classical voxelwise FDR versus LISA (motor task), sample size 20. The results on the left hand side of this figure were obtained using the classical algorithm of Benjamini-Hochberg (1995) applied at the voxel level. Here it is assumed that the tests are independent or positively correlated. For a side-by-side comparison, the results of LISA are shown, see Fig 2 of the manuscript. The difference between the two maps is shown below.

HCP Emotion task

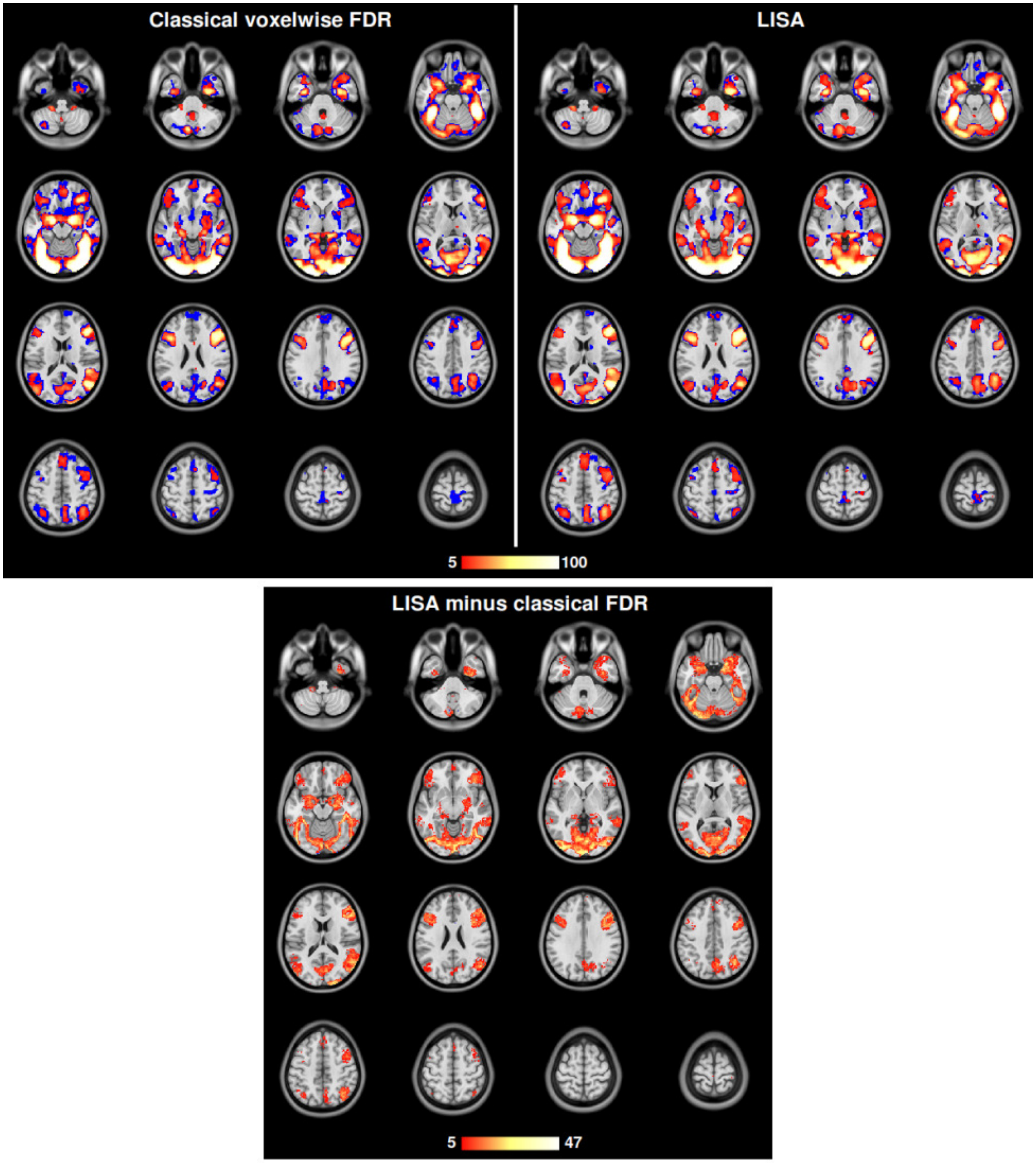

Supplementary Figure 20: Reproducibility maps, classical voxelwise FDR versus LISA (emotion task), sample size 20. The results on the left hand side of this figure were obtained using the classical algorithm of Benjamini-Hochberg (1995) applied at the voxel level. Here it is assumed that the tests are independent or positively correlated. For a side-by-side comparison, the results of LISA are shown, see Fig 2 of the manuscript. The difference between the two maps is shown below.

HCP Motor task

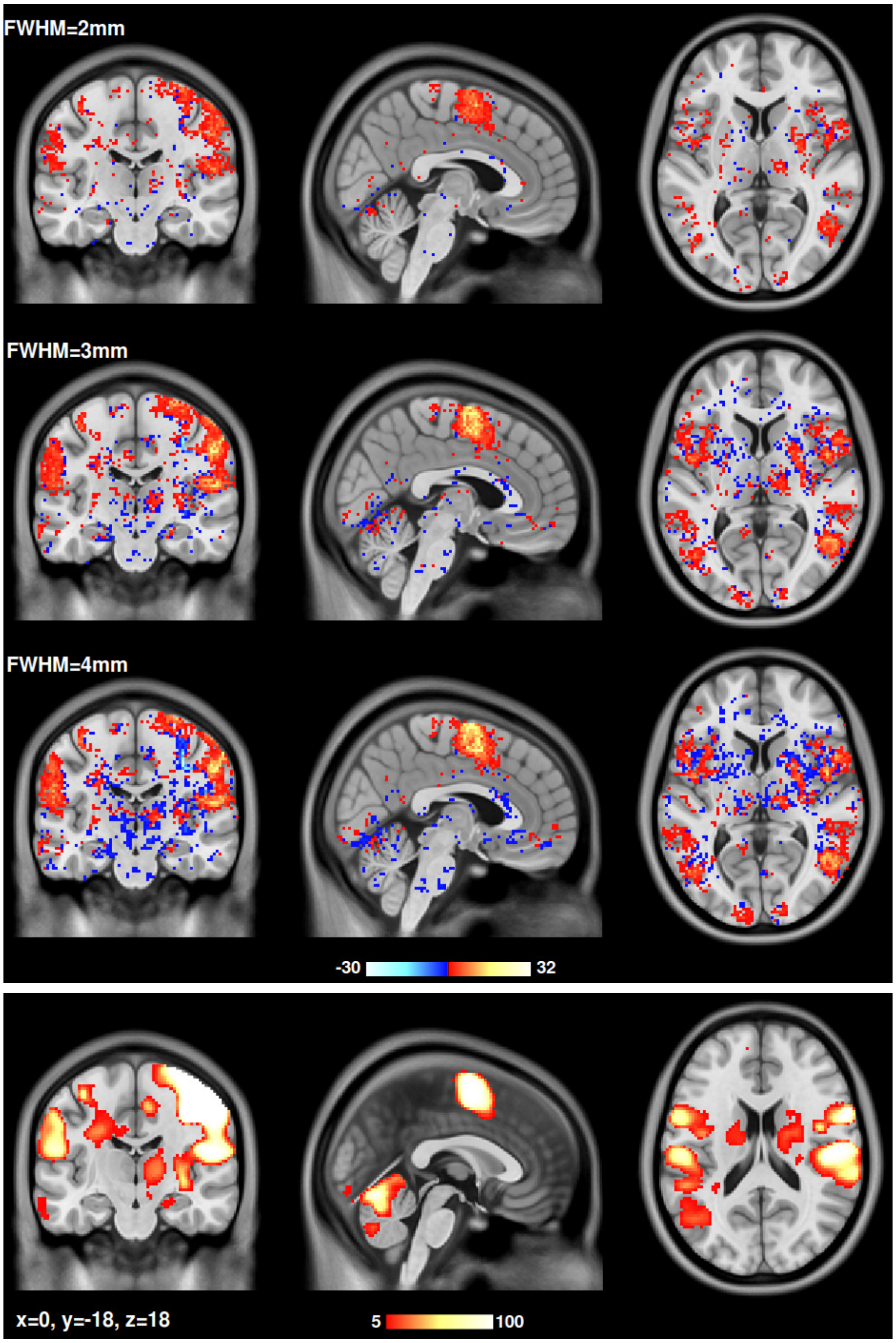

Supplementary Figure 21: Difference between reproducibility maps (motor task, sample size 20), Gaussfilter applied before versus after tests. The figure shows the effect of using a Gaussfilter applied before versus after the statistical test using various kernel sizes, see Fig 1 of the main manuscript. All data sets were preprocessed using a Gaussian filter with a smaller kernel size of FWHM=4mm (previously fwhm=6mm) to make room for the additional pre/post-test filtering. As a reference, the figure at the bottom row shows the reproducibility map obtained using FSL-TFCE (sample size 80). Note that the post-test Gaussfilter shows better reproducibility compared to the pre-test Gaussfilter particularly inside the main activation areas.

HCP Emotion task

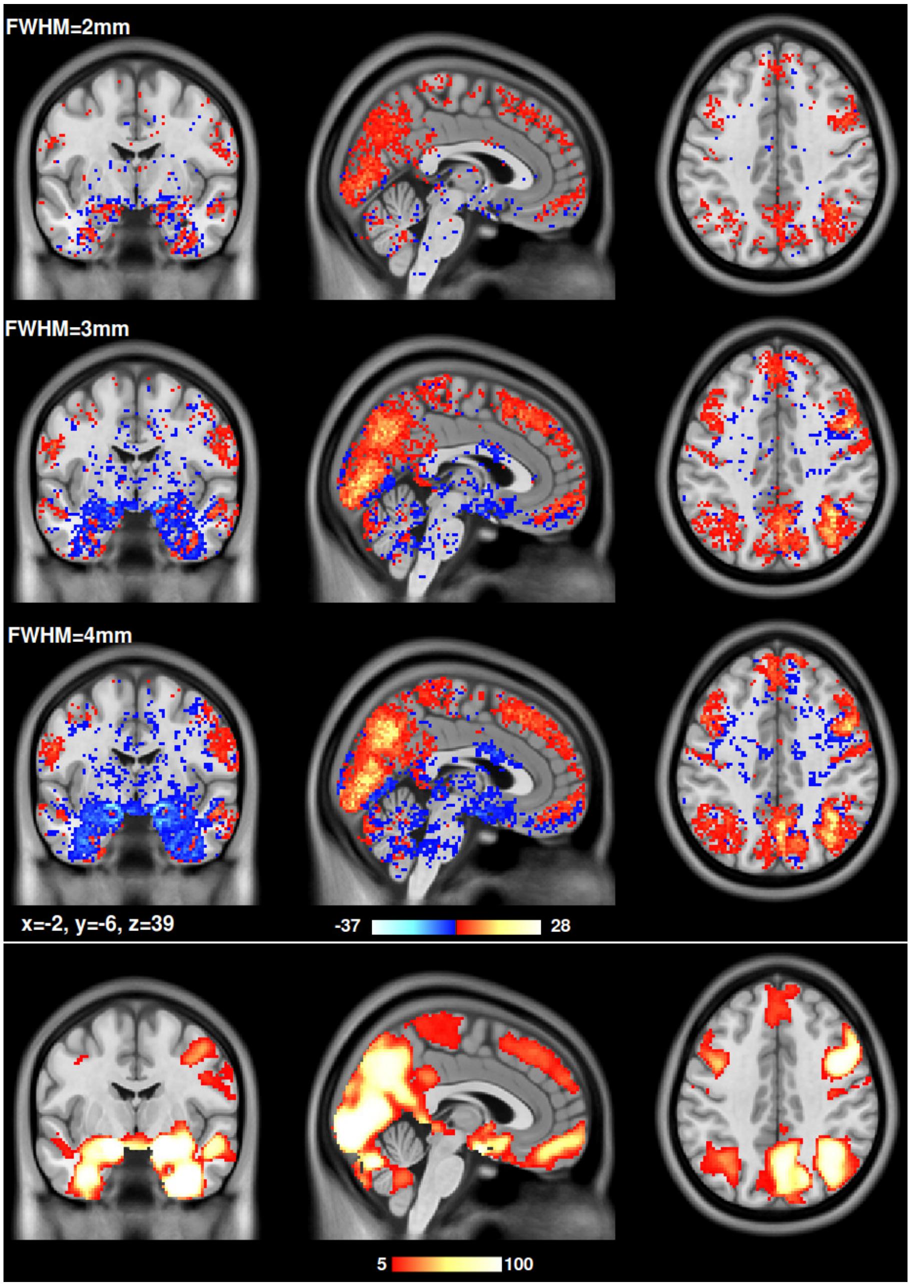

Supplementary Figure 22: Difference between reproducibility maps (emotion task, sample size 20), Gaussfilter applied before versus after tests. The figure shows the effect of using a Gaussfilter applied before versus after the statistical test using various kernel sizes, see Fig 1 of the main manuscript. All data sets were preprocessed using a Gaussian filter with a smaller kernel size of FWHM=4mm (previously fwhm=6mm) to make room for the additional pre/post-test filtering. As a reference, the figure at the bottom row shows the reproducibility map obtained using FSL-TFCE (sample size 80). Note that the post-test Gaussfilter shows better reproducibility compared to the pre-test Gaussfilter particularly inside the main activation areas.

HCP Motor task

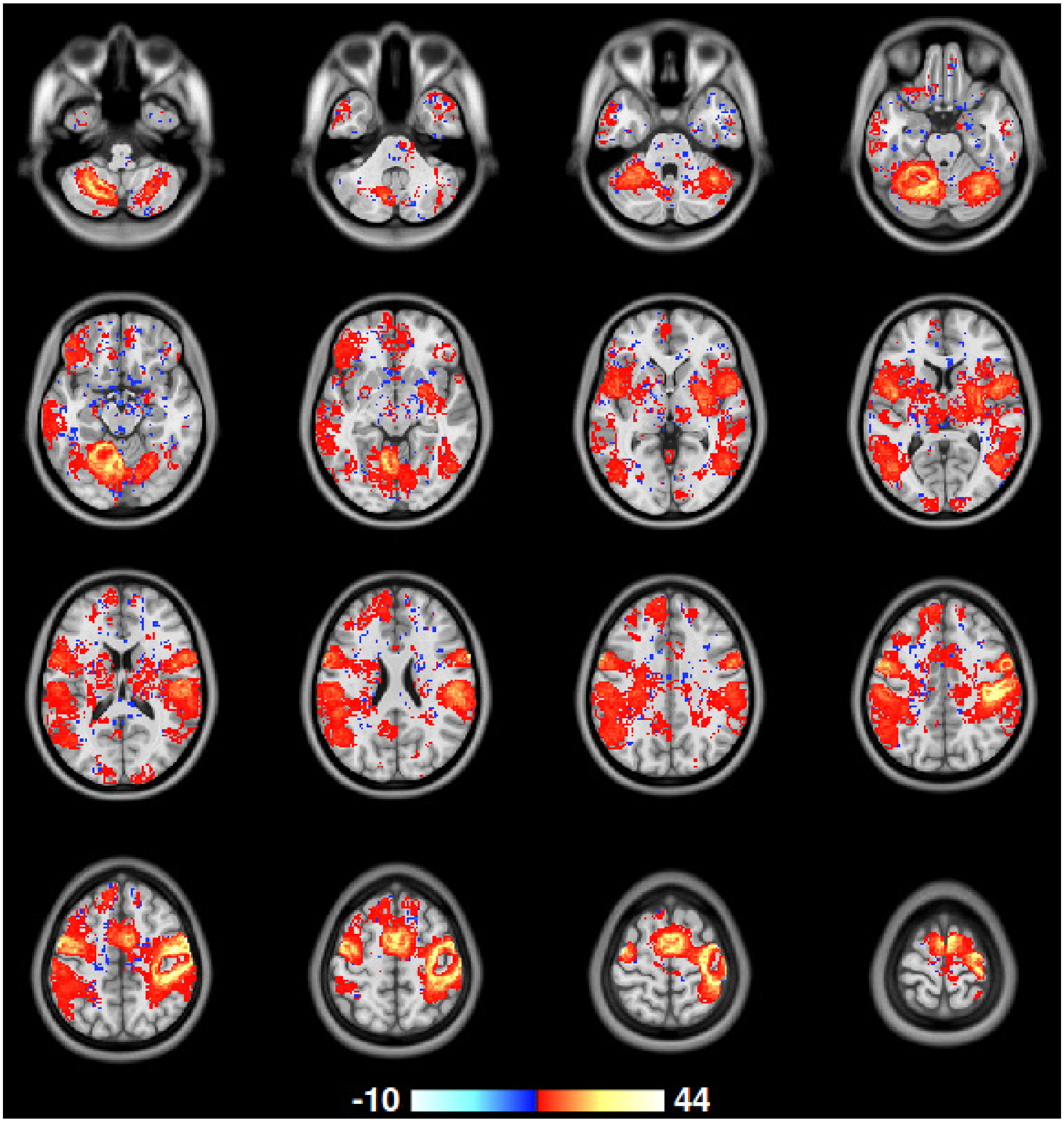

Supplementary Figure 23: Difference in reproducibility, bilateral filter minus no filter (motor task). The image shows the difference between two reproducibility maps across 100 tests based on randomly drawn samples of size twenty. Reproducibility was assessed either with or without filtering. The reproducibility maps were computed in the same way as in Fig 2 of the manuscript. The colors represent differences in reproducibility scores. For example, a value of 20 means that this voxel survived FDR thresholding in 20 more tests with bilateral filtering than without. Thus, the bilateral filter produces much better reproducibility.

HCP Emotion task

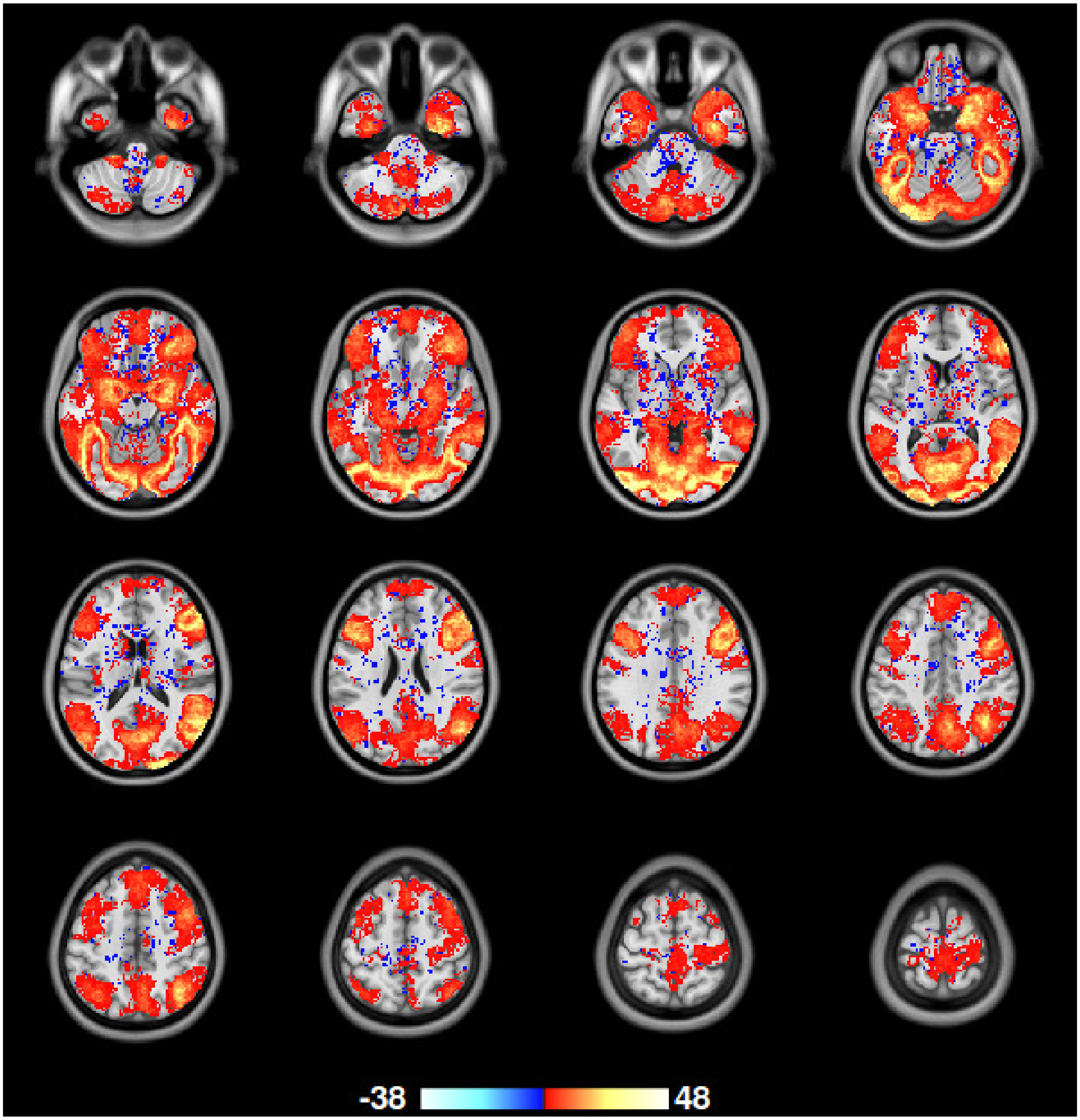

Supplementary Figure 24: Difference in reproducibility, bilateral filter minus no filter (emotion task). The image shows the difference between two reproducibility maps across 100 tests based on randomly drawn samples of size twenty. Reproducibility was assessed either with or without filtering. The reproducibility maps were computed in the same way as in Fig 2 of the manuscript. The colors represent differences in reproducibility scores. For example, a value of 20 means that this voxel survived FDR thresholding in 20 more tests with bilateral filtering than without. Thus, the bilateral filter produces much better reproducibility.

Simulations for comparing filters

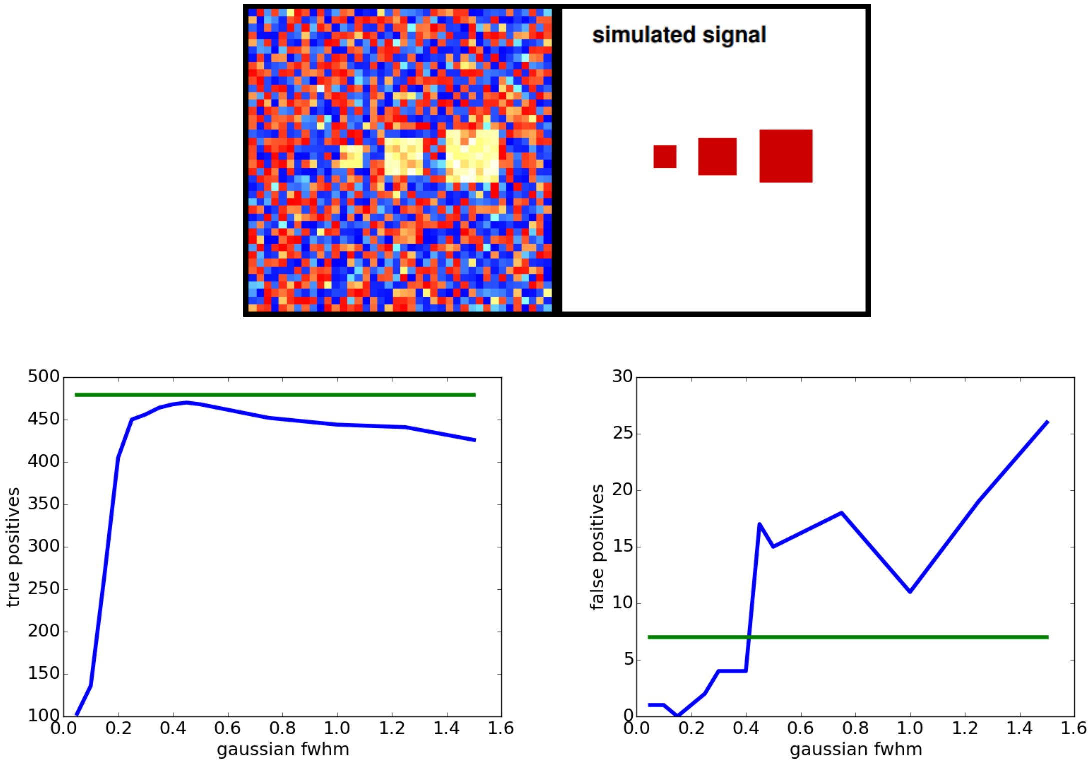

Supplementary Figure 25: Bilateral filter versus Gaussfilter. The blue lines show the true positive (TP) and false positive (FP) values found for the Gaussfilter at various kernel sizes. The numbers denote voxels. The green lines in the two figures show the TP and FP values found for the bilateral filter. Note that the TP values are consistently lower for the Gaussfilter than for the bilateral filter. The FP values of the Gaussfilter are only lower at kernel sizes for which the TP values are poor. Hence, the bilateral filter outperforms the Gaussfilter across all kernel sizes.

Supplementary Methods 5: Simulations for power estimation

Here we describe a method for generating synthetic data to be used for power calculations. The first step is to fill an image with spatially uncorrelated random Gaussian noise. Next, this image is spatially convolved with the autocorrelation function (ACF) that was recently proposed by Cox et al. [16, p. 156]

It is defined as

$$ACF(r) \; = \; a\,exp(-r^2/2b^2) \, + \, (1-a)\,exp(-r/c)$$

with $0 \leq a \leq 1$, $b > 0$, $c > 0$, and r the Euclidean distance between voxels. For the simulations, we have used four sets of parameters, see Supplementary Table 3 below. These values resulted from linear interpolation of values obtained via a personal communication by Dr. Robert Cox, NIMH, Bethesda, MD. Below, the ACF at a=0.8, b=4.33, c=16.76 is shown (Supplementary Figure 26). Note that the new ACF model shows a "heavy tail" which sets it apart from the Gaussian model.

We then added synthetic signals using signal-to-noise ratios in the range of $[0.4, 1.0]$. The signals had various shapes and sizes, see Supplementary Figure S19. For each simulation run, we generated 26 maps to simulate a typical sample size of fMRI group studies. For more about typical sample sizes, see Poldrack et al (2017, Nat Rev Neurosci, 18:115-126) Finally, we performed statistical inferences using the same four methods as in figs 2,3 of the main manuscript (SPM, FSL-TFCE, AFNI and LISA), and calculated their sensitivity. We repeated these experiments three times with varying background noise patterns.

	S1	S2	S3	S4
a	1.0	0.9	0.8	0.7
b	1.86	3.09	4.33	5.56
c	18.74	17.76	16.76	15.77

Supplementary Table 3: Parameter settings for the ACF model.

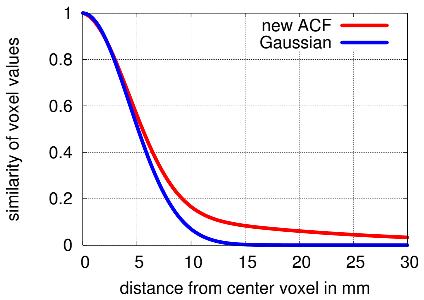

Supplementary Figure 26: The spatial autocorrelation (ACF) using a=0.8, b=4.33, c=16.76

Simulating spatial autocorrelation

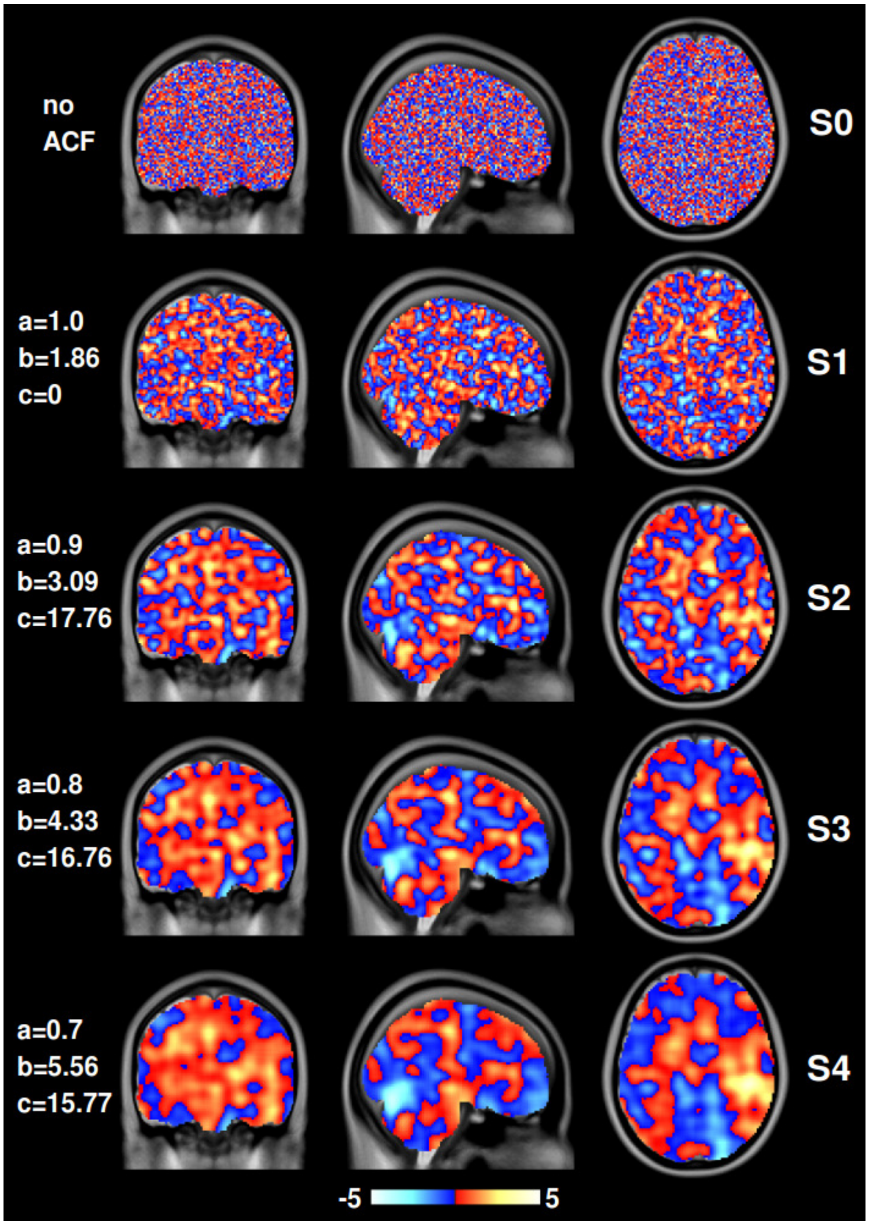

Supplementary Figure 27: Procedure for generating noise with a given spatial autocorrelation (ACF). The ACF model used here is defined as $ACF(r) = a\,exp(-r^2/2b^2) + (1-a)\,exp(-r/c)$ (Cox et al, 2017, Brain Connectivity 7(3):152-171). The top image (S0) was generated by filling a brain template with spatially uncorrelated random Gaussian noise. The other images (S1,...S4) were generated by convolving the top image with the ACF using the parameters shown on the left. Note that spatial smoothness increases from S1 to S4. An arbitrary number of such noise fields can be generated by varying the seed of the random number generator used for generating S0. The underlying brain template has 221336 voxels and simulates a spatial resolution of $(2mm)^3$.

Artificial signals

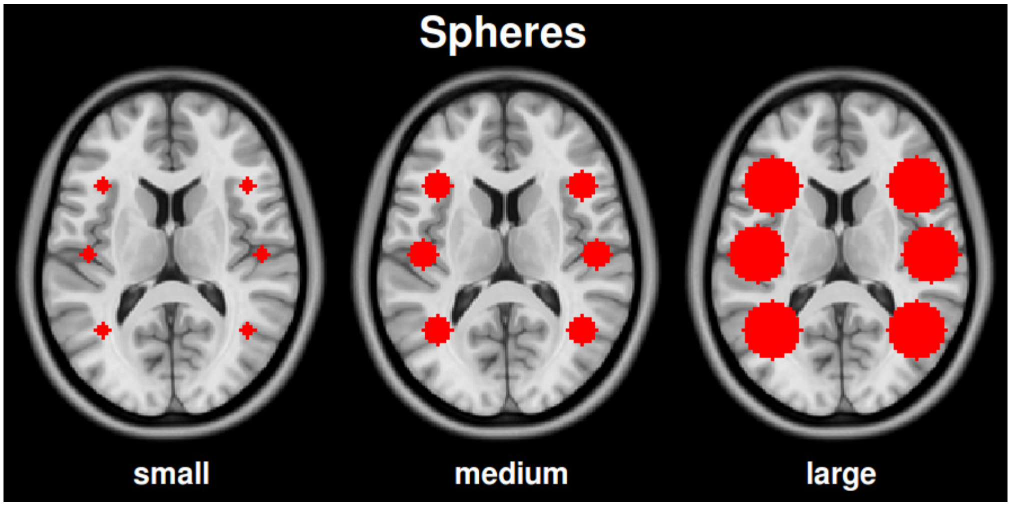

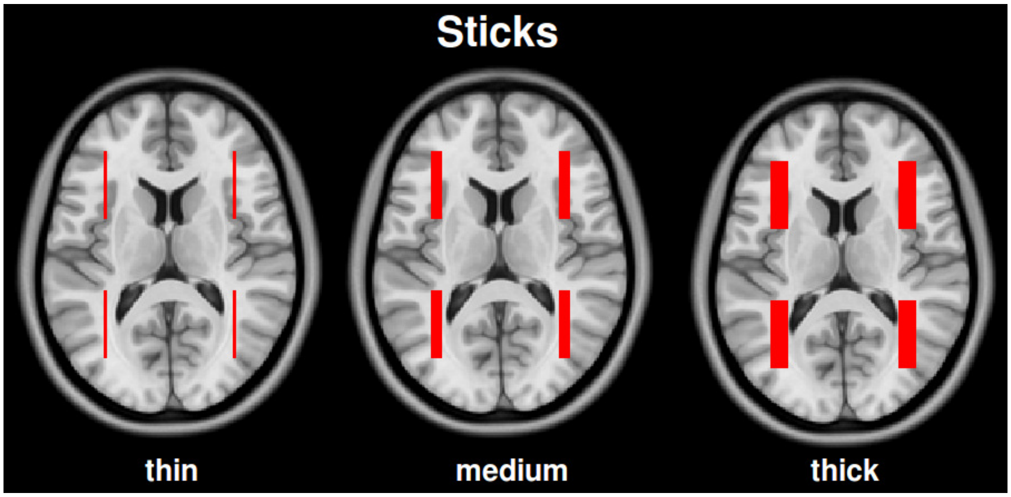

Supplementary Figure 28: Artificial signals. Three different sphere sizes were used: 33 voxels (small), 257 (medium), 2109 (large). The stick shapes vary in thickness. Their dimensions are (in voxels): 1x1x19=19 (thin), 3x3x19=171 (medium), 5x5x19=475 (thick).

Results of power estimation (spheres)

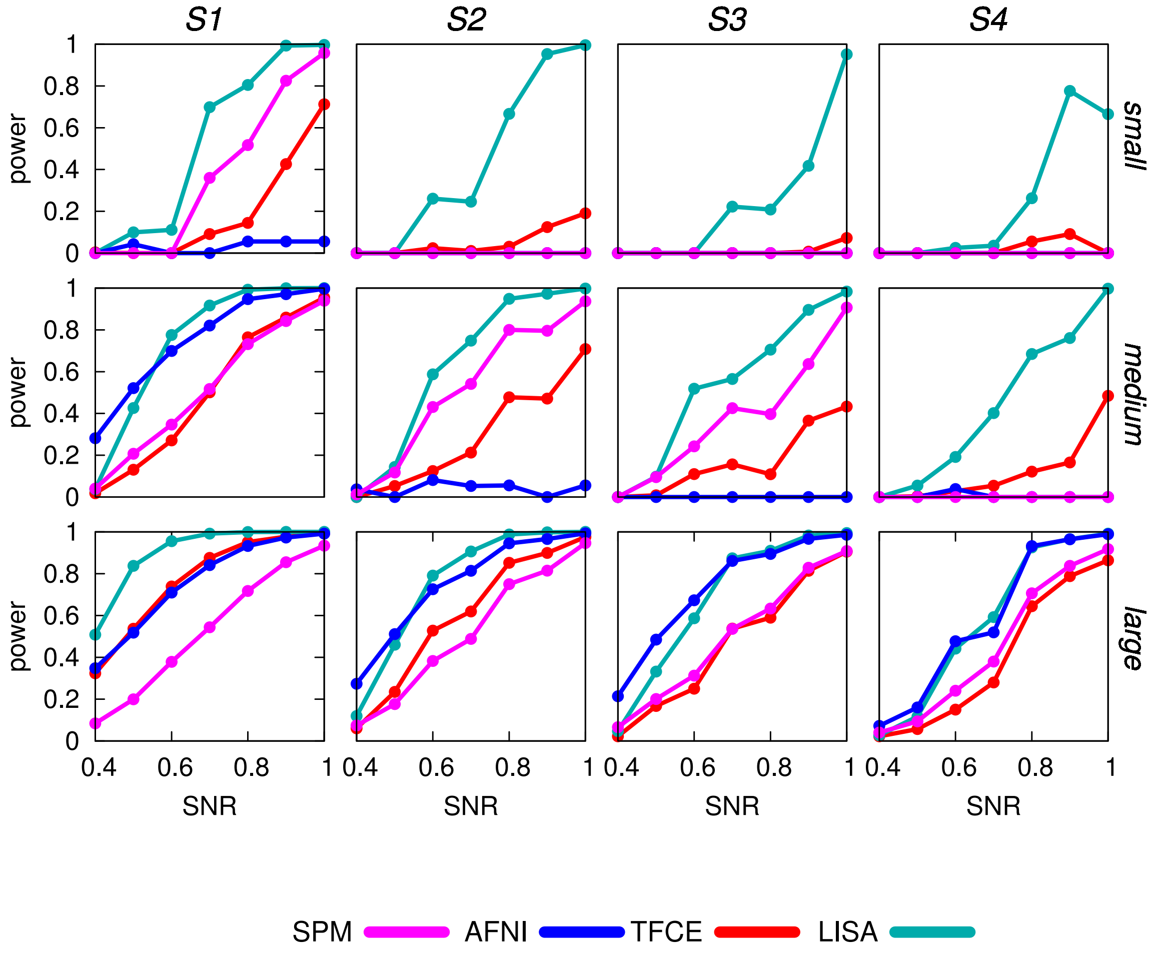

Supplementary Figure 29: Power estimation based on simulations using spheres as signals. Results obtained from small spheres are shown in the top row, medium sized spheres in the middle row, large spheres in the bottom row. Four levels of spatial smoothness (S1,...S4) as shown in Supplementary Figure 26 were used. The signal to noise ratio varied from SNR=0.4 to SNR=1.0. Statistical inference was performed using four methods: SPM-GRF, AFNI, FSL-TFCE, and LISA. SPM and AFNI require initial cluster defining thresholds. They were set to CDT=0.001 (SPM) and CDT=0.01 (AFNI). As expected, sensitivity increased for all methods with increasing sphere size and increasing SNR. All methods performed poorly at SNR=0.4. Compared to the other methods, LISA shows higher power for small spheres, and for medium sized spheres added to smooth backgrounds (S3,S4). The advantage is less pronounced for large spheres presented against smooth backgrounds (S2,S3,S4). The data presented here are an average across three runs. The results of each single run are shown in Supplementary Figure 31.

Results of power estimation (sticks)

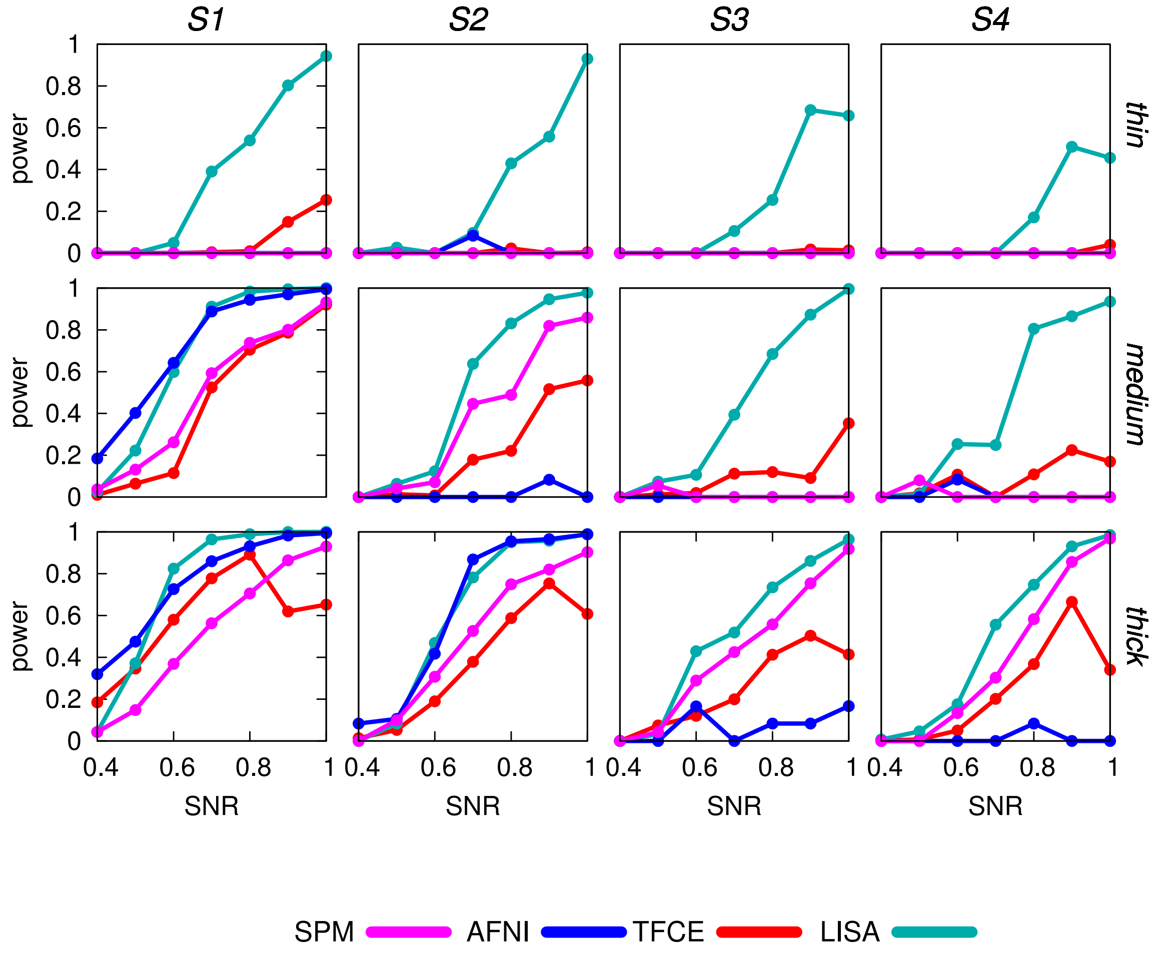

Supplementary Figure 30: Power estimation based on simulations using sticks as signals. Results obtained from thin sticks are shown in the top row, medium sticks in the middle row, thick sticks in the bottom row. Four levels of spatial smoothness (S1,..,S4) as shown in Supplementary Figure 26 were used. The signal to noise ratio varied from SNR=0.4 to SNR=1.0. Statistical inference was performed using four methods: SPM-GRF, AFNI, FSL-TFCE, and LISA. SPM and AFNI require initial cluster defining thresholds. They were set to CDT=0.001 (SPM) and CDT=0.01 (AFNI). As with the sphere shapes, sensitivity increased for all methods with increasing shape size and increasing SNR, and all methods performed poorly at SNR=0.4. Compared to the other methods, LISA shows higher power for thin sticks, and medium sized sticks added to smooth background noise (S3,S4). The advantage is less pronounced for large shapes presented against non-smooth backgrounds (S1,S2). The data presented here are an average across three runs. The results of each single run are shown in Supplementary Figure 31.

Power estimation, all three runs (spheres)

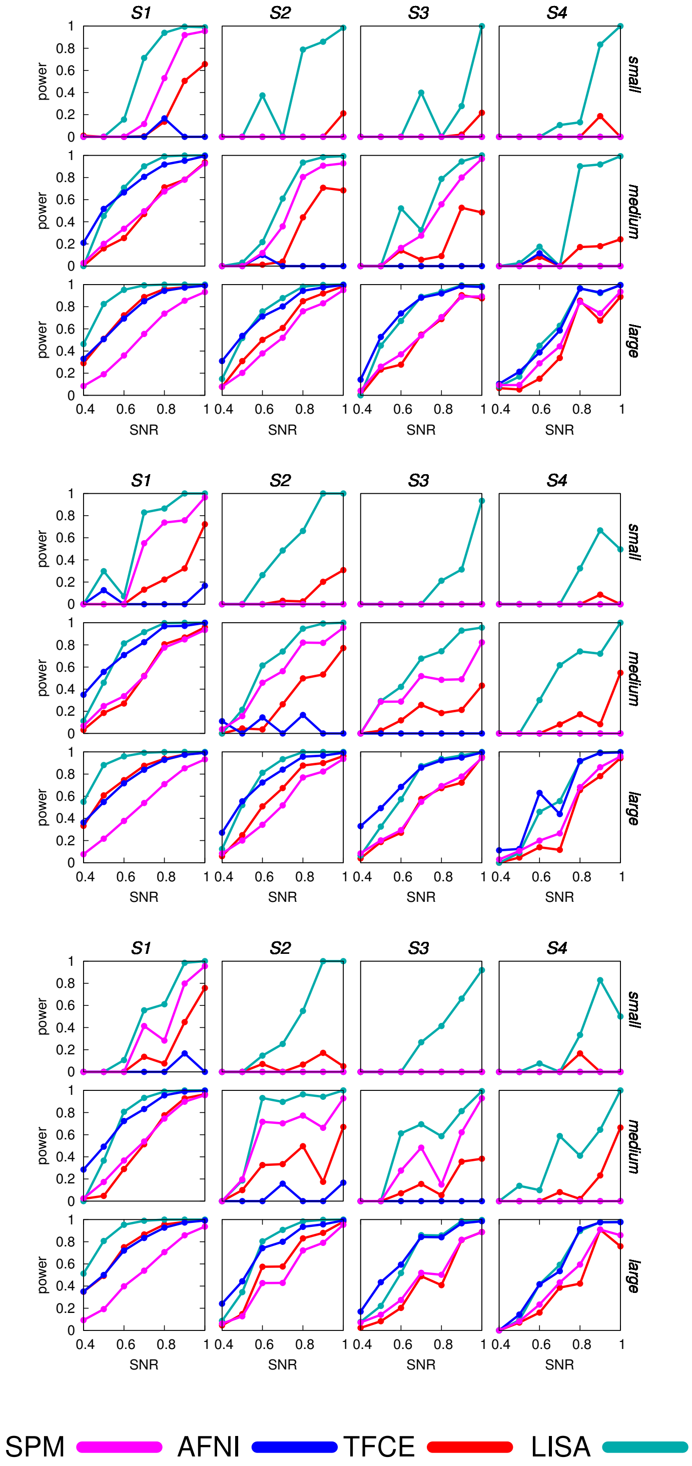

Supplementary Figure 31: Power estimation based on simulations using spheres as signals. The results all all three runs are shown.

Power estimation, all three runs (sticks)

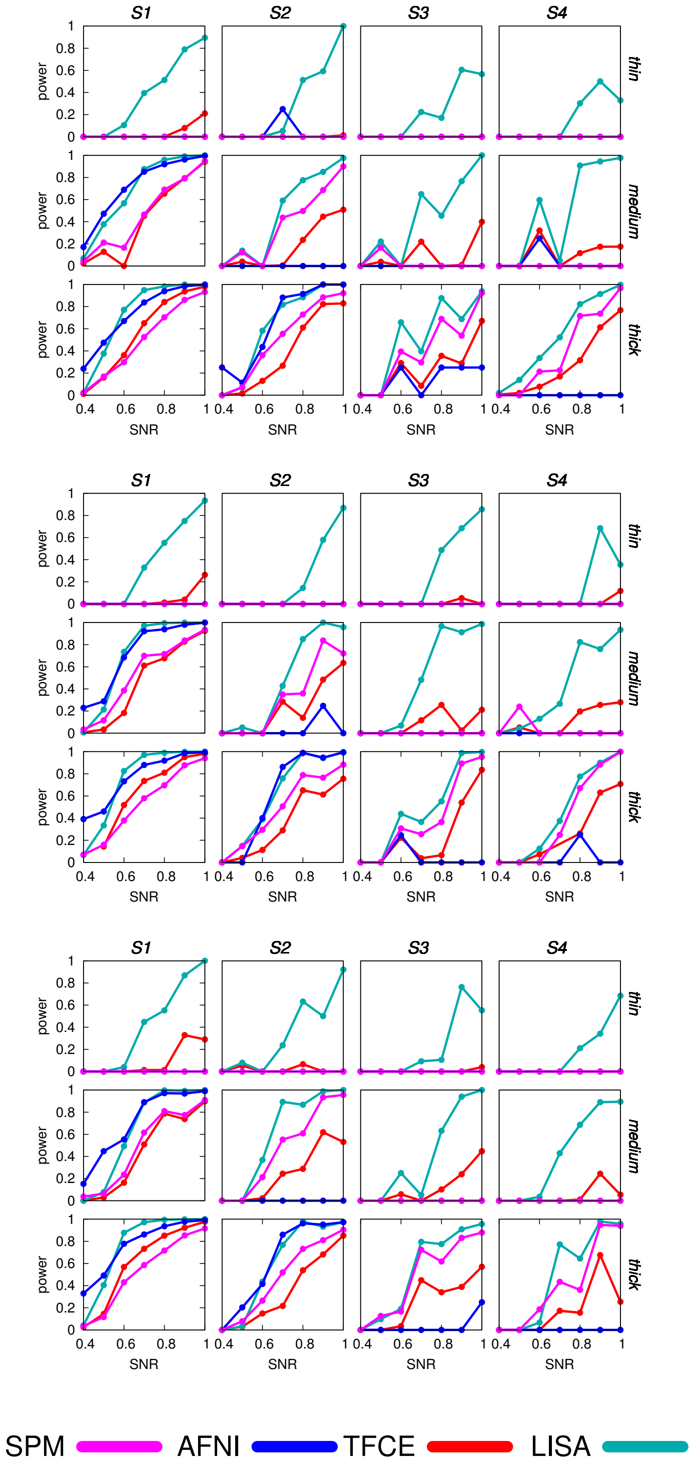

Supplementary Figure 32: Power estimation based on simulations using sticks as signals. The results all all three runs are shown.

Supplementary references

[1] Eklund, A., Nichols, T. & Knutsson, H. Cluster failure: Why fMRI inferences for spatial extent have inflated false-positive rates. *PNAS* **113**, 7900–7905 (2016).

[2] SPM8 (2009). `www.fil.ion.ucl.ac.uk/spm/software/spm8`.

[3] Eklund, A., Nichols, T., Andersson, M. & Knutsson, H. Empirically investigating the statistical validity of SPM, FSL and AFNI for single subject fMRI analysis. In *[IEEE 12th International Symposium on Biomedical Imaging (ISBI)* (2015).

[4] Van Essen, D. *et al.* The WU-Minn Human Connectome Project: An overview. *Neuroimage* **80**, 62–79 (2013).

[5] Glasser, M. *et al.* The human connectome project's neuroimaging approach. *Nature Neuroscience* **19**, 1175–1187 (2016).

[6] Glasser, M. *et al.* The minimal preprocessing pipelines for the Human Connectome Project. *Neuroimage* **80**, 105–124 (2013).

[7] Major Issue with preprocessed 7T fMRI data. www.humanconnectome.org/study/hcp-young-adult/news/major-issue-preprocessed-7t-fmri-data (2017).

[8] Griffanti, L. *et al.* ICA-based artefact removal and accelerated fMRI acquisition for improved resting state network imaging. *NeuroImage* **95**, 232–247 (2014).

[9] Salimi-Khorshidi, G. *et al.* Automatic denoising of functional MRI data: combining independent component analysis and hierarchical fusion of classifiers. *NeuroImage* **90**, 449–468 (2014).

[10] Glover, G. Deconvolution of impulse response in event-related BOLD fMRI. *Neuroimage* **9**, 416–419 (1999).

[11] Barch, D. *et al.* Function in the human connectome: task-fMRI and individual differences in behavior. *Neuroimage* **80**, 169–189 (2013).

[12] Smith, S. *et al.* Advances in functional and structural MR image analysis and implementation as FSL. *Neuroimage* **23**, S208–S219 (2004).

[13] Jenkinson, M., Beckmann, C., Behrens, T., Woolrich, M. & Smith, S. FSL. *NeuroImage* **62**, 782–90 (2012).

[14] Woolrich, M. *et al.* Bayesian analysis of neuroimaging data in FSL. *Neuroimage* **45**, S173–S186 (2009).

[15] Kuehn, E., Haggard, P., Villringer, A., Pleger, B. & Sereno, M. Visually-driven maps in area 3b. *Journal of Neuroscience* **38**, 1295–1310 (2018).

[16] Cox, R., Reynolds, R. & Taylor, P. FMRI Clustering in AFNI: and False Positive Rates Redux. *Brain Connectivity* **7** (2017).
